# Assessing the quality of reports about randomized controlled trials of scalp acupuncture combined with another treatment for stroke

**DOI:** 10.1186/s12906-017-1950-6

**Published:** 2017-09-06

**Authors:** Young-Nim You, Myung-Rae Cho, Jae-Hong Kim, Ju-Hyung Park, Gwang-Cheon Park, Min-Yeong Song, Jin-Bong Choi, Jae-Young Han

**Affiliations:** 1 Clinical Research Center, DongShin University Gwangju Oriental Hospital, Gwangju City, Republic of Korea; 2 Department of Acupuncture and Moxibustion Medicine, College of Korean Medicine Dong-Shin University, Naju City, Republic of Korea; 3 Department of Korean Rehabilitation Medicine, College of Korean Medicine Dong-Shin University, Naju City, Republic of Korea; 4 Department of Physical & Rehabilitation Medicine, Chonnam National University Medical School and Hospital, Gwangju City, Republic of Korea

**Keywords:** Randomized controlled trials, Scalp acupuncture, Stroke, Consort, STRICTA

## Abstract

**Background:**

This study was designed to assess the quality of reporting on randomized controlled trials (RCTs) of scalp acupuncture for the treatment of stroke.

**Methods:**

The following 8 databases were systematically investigated from their inception to December 2015: PubMed, Embase, Cochrane Library, China National Knowledge Infrastructure, National Institute of Informatics Scholarly and Academic Information Navigator, National Digital Science Library, Korean Traditional Knowledge Portal, and Korean Studies Information Service System. RCTs utilizing scalp acupuncture as an intervention for stroke were selected, and the quality of reports was assessed based on the Consolidated Standards of Reporting Trials 2010 statement (CONSORT) and Standards for Reporting Interventions in Controlled Trials of Acupuncture 2010 (STRICTA). For each study, the overall quality score (OQS) of 13 CONSORT items, a combined key methodological index score (MIS) of 5 CONSORT items, and the OQS of 17 STRICTA items were measured.

**Results:**

The original reports of 63 RCTs were ultimately obtained, and the median CONSORT OQS was 7 (minimum 2, maximum 11). Particularly, the items ‘trial design’, ‘sample size’, ‘ancillary analyses’, and ‘harms’ had a positive rate of less than 10%. The median MIS was 1 (minimum 0, maximum 5), with ‘allocation concealment and implementation’ and ‘intent-to-treat analysis (ITT) analysis’ having a positive rate of less than 10%. The median STRICTA OQS was 11 (minimum 6, maximum 14), and only the items ‘sample size’ and ‘intent-to-treat analysis’ were reported, with a positive rate of less than 10%. The mean CONSORT OQS increased by approximately 0.81 for each 5-year period in which manuscripts were published (95% confidence interval: 0.43 to 1.19; *p* < 0.001). No variable was significantly associated with MIS in the ordinal regression model.

**Conclusion:**

The quality of reports on RCTs investigating scalp acupuncture treatment for stroke was moderate to low. Furthermore, reporting of some items was either insufficient or inadequate in the majority of studies. In order to improve and standardize the quality of RCTs investigating scalp acupuncture for stroke, CONSORT and STRICTA guidelines should be utilized more frequently.

**Electronic supplementary material:**

The online version of this article (10.1186/s12906-017-1950-6) contains supplementary material, which is available to authorized users.

## Background

Stroke is one of the three leading causes of death in China as well as in western countries [1–3]. In Korea, mortality due to stroke is 80 per 100,000 people, with 25,000 deaths attributed to cerebrovascular diseases including stroke in 2014 alone [1]. Globally, stroke causes 6.2 million mortalities per year and is the second leading cause of death after ischemic heart disease including heart failure [4]. Once stroke occurs, its mortality rate is high, and the likelihood of patients returning to active social life is low; therefore, its socio-economic impact is also high [5].

Scalp acupuncture is a modality that treats diseases of the entire body by placing a needle on a corresponding area of the scalp based on the functional principle of cerebral cortex location and the Standard International Acupuncture Nomenclature (SIAN) proposed by World Health Organization, which were developed in 1991 [6–8]. Scalp acupuncture has proven effective for the treatment of cerebrovascular diseases, neurodegenerative disorders, and diseases of the central nervous system [6, 9–12]. Furthermore, a number of clinical trials have reported therapeutic effects of scalp acupuncture for the treatment of stroke [13–15].

Randomized controlled trials (RCTs) are considered the optimal study design to examine the therapeutic effects and efficacy of scalp acupuncture [16]. However, even with an RCT design, inappropriate study methodology can affect the reliability and validity of the results and thus the quality of its findings [17]. Therefore, it is necessary to evaluate the quality of RCTs based on systematic quality control standards and assessment of their design, implementation, and analysis [18].

The Consolidated Standards of Reporting Trials (CONSORT), developed in 2001 and revised in 2010, provide guidelines to improve clinical trial reporting in order to identify biased results, with the purpose of facilitating the assessment and interpretation of RCTs [19]. The STandards for Reporting Interventions in Controlled Trials of Acupuncture (STRICTA) were developed in 2001 and revised in 2010 to improve the reporting of acupuncture implemented in clinical trials [20]. The combination of these two guidelines can aid in the assessment of the completeness as well as transparency of RCTs [21].

The aims of this study were to assess the quality and limitations of articles reporting RCTs of scalp acupuncture treatment for stroke published through December 2015 by using CONSORT and STRICTA, and to further promote improvement in the quality of future clinical trials.

## Methods

### Literature search methods

The following 8 databases were searched from their inception through December 2015 for published articles and databases: PubMed, Embase, the Cochrane Library, China National Knowledge Infrastructure (CNKI), National Institute of Informatics Scholarly and Academic Information Navigator (CiNii), National Digital Science Library (NDSL), Korean Traditional Knowledge Portal (KTKP), and Korean Studies Information Service System (KISS). The terms “stroke”, “hemiplegia”, “cerebral”, “infarction”, “cerebral infarction”, “cerebrovascular”, “apoplexy” were searched in combination with each of the following: “acupuncture”, “scalp acupuncture”, “head acupuncture”, “skull acupuncture”, “brain acupuncture”, “cerebral acupuncture”, “cranial acupuncture”. No limits were applied for language and country.

### Literature selection and exclusion criteria

#### Types of studies

We looked at RCTs that assessed the effect of a scalp acupuncture treatment in stroke patients. Non-randomized, cross-over RCTs, case reports, and case-control studies were excluded.

#### Types of participants

All study subjects with a clinical diagnosis of acute and chronic stroke were included regardless of age, sex, or other demographic factors. Stroke was diagnosed according to the Chinese Medical Association diagnostic standards [22–24] or confirmed by magnetic resonance imaging (MRI) or computed tomography (CT).

#### Types of interventions

Interventions consisting of a combination of scalp acupuncture treatment with another acupuncture modality (e.g., electro-acupuncture, body acupuncture, and ear acupuncture), and western medicine, Chinese herbal medicine, and rehabilitation were included.

### Evaluation of report quality

#### Rating of overall reporting quality

For overall quality score (OQS) based on the CONSORT 2010 guidelines, 13 items (range, 0 to 13) were graded [25]. The items in the CONSORT discussion session were excluded, as they involved subjective evaluation (Table 1). Seventeen items based on the STRICTA guidelines (range, 0 to 17) were also graded (Table 2) [21]. To grade the quality of reports, 1 point was assigned if information for each item was stated, and 0 points were assigned if the item was not addressed or uncertain.

**Table 1:** Overall quality score of reporting using items from the CONSORT statement (*n* = 63)

Item	Criteria	Description	Number of positive trials	%	Cohen’s *к* coefficient	95% CI
1	‘Randomized’ in The title or abstract	Study identified as a randomized controlled in the title or abstract	63	100	1.00	1.00
2	Background	Adequate description of the scientific background and explanation of rationale	17	27	0.82	0.66 to 0.97
3	Trial design	Description of trial design (such as parallel, factorial) including allocation ratio	4	6	1.00	1.00
4	Participants	Description of the eligibility criteria for participants	50	79	0.58	0.28 to 0.87
5	Interventions	Details of the interventions intended for each group	54	86	0.77	0.52 to 1.02
6	Outcomes	Definition of primary (and secondary when appropriate) outcome measures	49	78	0.79	0.60 to 0.99
7	Sample size	Description of sample size calculation	1	2	1.00	1.00
12	Statistical methods	Description of the statistical methods used to compare groups for primary outcomes, subgroup analyses, or adjusted analyses	49	78	0.85	0.69 to 1.02
13	Flow chart	Details on the flow of participants through each stage of the trials (number of patients randomly assigned, receiving intended treatment, completing the protocol and analyzed)	59	84	0.85	0.56 to 1.14
14	Recruitment	Dates defining the periods of recruitment and follow-up	38	60	0.59	0.37 to 0.81
17	Outcomes and estimation	For each primary and secondary outcome, a summary of results for each group is given, and the estimated effect size and its precision (for example, 95% CI)	53	84	0.71	0.44 to 0.99
18	Ancillary analyses	Clear statement of whether subgroup/adjusted analyses were prespecified or exploratory	0	0	1.00	1.00
19	Harms	Description of all important adverse events in each group	2	3	1.00	1.00

**Table 2:** Overall quality score of reporting using items from STRICTA guidelines (*n* = 63)

Item	Criteria	Description	Number of positive trials	%	Cohen’s *к* coefficient	95% CI
1	Acupuncture rationale	(1a) Style of acupuncture (e.g., Traditional Chinese Medicine, Japanese, Korean, Western medical, Five Element, ear acupuncture, etc.)	63	100	1.00	1.00
		(1b) Reasoning for treatment provided, based on historical context, literature sources and/or consensus methods, with references where appropriate	56	89	0.82	0.56 to 1.02
		(1c) Extent to which treatment was varied	2	3	0.79	0.64 to 1.20
2	Details of needling	(2a) Number of needle insertions per subject per session (mean and range where relevant)	9	14	0.83	0.64 to 1.02
		(2b) Names (or location if no standard name) of points used (uni−/bilateral)	63	100	1.00	1.00
		(2c) Depth of insertion, based on a specified unit of measurement Or on a particular tissue level	37	59	0.57	0.36 to 0.79
		(2d) Responses sought (e.g., de qi or muscle twitch response)	52	83	0.88	0.72 to 1.04
		(2e) Needle stimulation (e.g., manual or electrical)	31	49	0.90	0.80 to 1.01
		(2f) Needle retention time	56	89	0.82	0.56 to 1.07
		(2 g)Needle type (diameter, length and manufacturer or material)	49	78	0.74	0.52 to 0.96
3	Treatment regimen	(3a) Number of treatment sessions	60	95	0.85	0.56 to 1.10
		(3b) Frequency and duration of treatment sessions	58	92	0.73	0.37 to 1.10
	Other components Of treatment	(4a) Details of other interventions administered to the acupuncture group (e.g., moxibustion, cupping, herbs, exercises, lifestyle advice)	35	56	0.58	0.37 to 0.79
		(4b) Setting and context of treatment, including instructions to practitioners, and information and explanations to patients	2	3	0.79	0.39 to 1.20
5	Practitioner background	(5) Description of participating acupuncturists (qualification or professional affiliation, years in acupuncture practice, other relevant experience)	35	56	0.70	0.51 to 0.88
6	Control or comparator interventions	(6a) Rationale for the control or comparator in the context of the research question, with sources that justify the choice(s)	22	35	0.56	0.36 to 0.76
		(6b) Precise description of the control or comparator. If sham acupuncture or any other type of acupuncture-like control is used, provide details as for items 1–3 above	48	76	0.63	0.37 to 0.89

#### Rating of key methodological items

Because five major methodological items (‘randomization’, ‘allocation concealment’, ‘blinding’, ‘baseline characteristics’, and ‘ITT analysis’) included in the CONSORT 2010 guidelines were related to potential factors causing bias, they were evaluated separately for each study (Table 3) [26–28]. One point was assigned for each item that was reported, and 0 points were assigned if the item was not reported or uncertain (range, 0 to 5).

**Table 3:** Reporting quality of key methodological items (*n* = 63)

Item	Criteria	Description	Number of positive trials	%	Cohen’s *к* coefficient	95% CI
8	Randomization	Description of the method used to generate the random sequence	19	30	0.79	0.63 to 0.95
9 and 10	Allocation concealment and implementation	Description of the method used to implement the random allocation sequence assuring the concealment until interventions are assigned	2	3	1.00	1.00
11	Blinding	Whether or not participants, those administering the interventions, or those assessing the outcomes were blinded to group assignment	6	10	0.91	0.75 to 1.08
15	Baseline data	An outline of baseline demographic and clinical characteristics of each group	13	21	0.68	0.47 to 0.89
16	Intent-to-treat analysis	No. of participants in each group included in each analysis and whether it was done by “intention to treat”	5	8	0.82	0.57 to 1.07

### Data extraction and analysis

Each report was independently evaluated by two reviewers (YNY and MYS) in reference to each item’s definition and detailed description in the CONSORT and STRICTA statements, and in cases of disagreement between these reviewers, final scores were determined through agreement with a third reviewer (MRC) (Tables 1, 2 and 3) [29].

Cohen’s *κ*-statistic was calculated in order to evaluate the degree of agreement between the two evaluators. A *κ* of 0.20 or lower was defined as ‘poor’ agreement, between 0.20 and 0.40 as ‘low’, between 0.40 and 0.60 as ‘moderate’, between 0.60 and 0.80 as ‘substantial’, and greater than 0.80 as ‘good’, with 1 representing perfect agreement [30]. Cohen’s *κ*-statistical analysis was performed using SAS software, version 9.3 (SAS Institute, Inc., Cary, NC, USA) [21, 25].

In order to evaluate the overall quality of reported RCTs and relevant factors, OQS was used as a dependent variable modeled using linear regression. Only variables with *p* ≤ 0.10 on univariate analysis were included in the multivariate regression model to identify significant variables (*p* ≤ 0.05). To analyze the factors related to methodological quality, MIS was used as an outcome variable in regression analysis. Linear and ordinal regression analysis was performed using SPSS software version 20.0 (SPSS, Chicago, IL, USA) [21, 25].

## Results

### Report selection

**Fig. 1:**
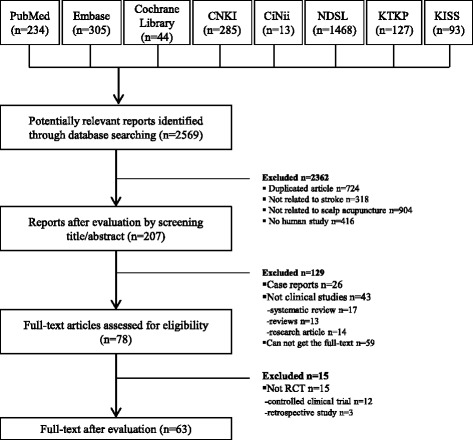
Flow chart of the article selection process

A total of 2569 relevant reports were identified, among which 207 reports related to the study topic were selected based on review of the title and abstract. A total of 63 relevant RCTs were ultimately extracted for final analysis (Additional file 1). The RCT selection process is outlined in Fig. 1.

### Characteristics of the reports included in this study

**Fig. 2:**
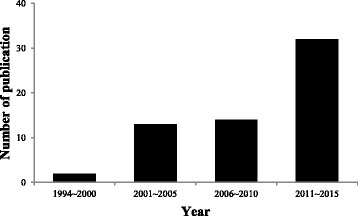
Number of publications

The 63 reports of RCTs evaluated in this study included 3 (4.8%) published prior to 2000, 14 (22.2%) published in 2001–2005, 14 (22.2%) published in 2006–2010, and 32 (50.8%) published in 2011–2015 (Fig. 2). The languages of the published articles included 1 in Korean (1.6%), 7 in English (11.1%), and 55 in Chinese (87.3%), and 60 articles (95%) were published in China by mainly Chinese authors. Control group interventions included 28 instances of western medication (44.4%), 14 instances of body acupuncture (22.2%), 3 instances of western medication with body acupuncture (4.8%), 2 instances of scalp electro acupuncture (3.2%), 2 instances of rehabilitation (3.2%), 2 instances of scalp acupuncture with rehabilitation (3.2%), 1 instance of scalp acupuncture with body acupuncture and rehabilitation (1.6%), 1 instance of body acupuncture with rehabilitation (1.6%), 1 instance of scalp acupuncture with body acupuncture (1.6%), 1 instance of scalp acupuncture with body acupuncture and western medication (1.6%), 1 instance of sham scalp acupuncture (1.6%), 1 instance of scalp acupuncture at the contralateral side (1.6%), 1 instance of scalp acupuncture with western medication (1.6%), 1 instance of scalp acupuncture with western medication and Chinese herbal medicine (1.6%), 1 instance of western medication with Chinese herbal medicine and rehabilitation (1.6%), 1 instance of ear acupuncture (1.6%), 1 instance of oral administration (1.6%), and 1 instance of Chinese herbal medicine (1.6%). The sample size of control groups ranged from 22 to 330 (Additional file 2).

### Evaluation of report quality

#### Rating of overall reporting quality using CONSORT

The graded quality of reports based on the CONSORT guidelines is presented in Table 1. The mean OQS was 7, ranging from 2 to 11 (Additional file 3). ‘Trial design’, ‘sample size’, ‘ancillary analyses’, and ‘harms’ were reported by fewer than 10% of articles, with information about these items insufficient or not articulated in most studies. Items 4 and 14 had a moderate degree of agreement; items 5, 6, and 17 had a substantial degree of agreement; items 2, 12, 13, and 14 had a good degree of agreement; and items 1, 3, 7, 18, and 19 had perfect agreement between reviewers (Table 1).

#### Rating of overall quality score using STRICTA

The results of the graded quality scoring of reports based on STRICTA guidelines are shown in Table 3. The median OQS was 11, ranging from 6 to 14 (Additional file 3). Items 1c and 4b were adequately reported by fewer than 10% of articles, ‘number of needle insertions’ (item 2a) was reported by 14%, and ‘rationale for the control or comparator’ (item 6a) was reported by 35%.

Items 2c, 4a, and 6a had a ‘moderate’ degree of agreement; items 1c, 2 g, 3b, 4b, 5, and 6b had a ‘substantial’ degree of agreement; items 1b, 2a, 2d, 2e, 2f, and 3a had a ‘good’ degree of agreement, and items 1a and 2b had a ‘perfect’ agreement between reviewers (Table 2).

#### Rating of key methodological items

The median MIS of five key methodological items based on the CONSORT guidelines was 1, ranging from 0 to 5 (Additional file 3). ‘Allocation concealment and implementation’ and ‘ITT analysis’ were reported by fewer than 10% of articles, and information was either insufficient or not articulated in the rest. Items 8 and 15 had a ‘substantial’ degree of agreement, items 11 and 16 had a ‘good’ degree of agreement, and item 9 and 10 had a ‘perfect’ agreement between reviewers (Table 3).

### Exploratory analysis: Factors associated with better reporting quality

On univariate analysis, year of publication was associated with increased OQS based on the CONSORT guidelines. After adjustment in the multivariate linear regression model, OQS significantly increased by approximately 0.81 for each 5-year period of manuscript publication (95% confidence interval: 0.43 to 1.19; *p* < 0.001) (Table 4). On univariate analysis, no factors were significantly related to OQS based on STRICTA guidelines. In the analysis of MIS, there was no statistically significant variable in the ordinal regression model (*p* > 0.05).

**Table 4:** Multivariate linear regression analysis for factors associated with better OQS based on the CONSORT statement (*n* = 63)

Variables	β	S.E.	t	*p*	95% CI
Constant	4.68	0.63	7.40	<0.001	3.41–5.94
Year of publication	0.812	0.19	4.28	<0.001	0.43–1.19

S.E.; standard error

## Discussion

The main strength of this study is that RCTs related to scalp acupuncture treatment were selected through a comprehensive and systematic search of 8 databases. Furthermore, the quality of RCTs included in this study was assessed by using the CONSORT and STRICTA guidelines. These two instruments were developed to identify issues underlying the unreliability of RCTs and have been broadly applied to assess the quality of reports in acupuncture research. Moreover, these guidelines are powerful tools, as evidenced by the fact that the majority of their items were well reported [31].

For the 63 RCTs included in this study, the median OQS for the reporting quality of CONSORT items was 7, out of a total of 13. For STRICTA items, the median OQS was 11, corresponding to approximately 60% of the total possible score of 17. However, reports of some items were found to be either inappropriate or insufficient in most of the examined studies, including ‘trial design’, ‘sample size’, ‘ancillary analyses’, and ‘harms’ among the CONSORT guidelines and ‘extent to which treatment was varied’ and ‘setting and context of treatment’ among the STRICTA guidelines.

For ‘trial design’, it is important to clearly articulate several aspects of RCT design (such as parallel, double-blind, placebo, and sham designs). Even if the same randomization ratio (such as 8:8 for two groups) was used, providing the allocation ratio is helpful in improving the quality of reports. Particularly in cases of atypical clinical trials requiring complicated analyses and interpretation or large sample sizes, the need to clearly establish the clinical design increases [32].

Sample size calculation is required for the statistical consideration of differences in therapeutic effects between a treatment group and a control group. A significant clinical difference between the intervention group and control group can only be detected reliably if the number of examinees is sufficient [21]. However, due to the challenge of collecting subjects meeting the study criteria within a certain period, it can be difficult to achieve the designed sample size [33, 34]. In addition, very small RCTs carry the risk of bias or may be insufficient for measuring a therapeutic benefit [29]. Therefore, in order to identify a significant difference with high reliability between the intervention and control groups, sample size determination should be the focus of increased attention through consultation with clinical statisticians.

‘Ancillary analyses’ refer to repeated analyses of the same data, which can become a source of bias through over-interpretation of the results [35], and reporting analysis results that have not been pre-established in the design of a clinical trial introduces bias through being selective [36]. Therefore, authors should report the results of analyses that have been predetermined to have high reliability, and clearly articulate the reason and purpose of any supplemental group analysis performed.

Although an RCT is the best method to generate efficacy and safety data, it is difficult to detect rare adverse events. Many RCTs present inappropriate [37] or low-quality reporting of adverse effects [38]. Additionally, the number of articles reporting severe adverse effects and information about subjects excluded from analysis due to adverse effects is very low [39]. However, in order for clinical trial participants to make a balanced and reasonable decision to participate, not only the benefits of the intervention but also information about its risks must be provided, and the existence and nature of adverse effects has a significant impact on whether a specific intervention can be considered allowable and useful [21].

The STRICTA item ‘extent to which treatment was varied’ (1c) was positively reported by only 3% of articles included in this study, similar to previous findings [40]. The variability of treatments in clinical trials must be minimized through standardized protocols, and the degree of personalized treatment should be discussed between a patient and their physician [41].

The STRICTA item ‘setting and context of treatment’ (4b) can also provide important additional components to treatment [42]. Because treatment by a physician or a change in the treatment situation of a patient can affect test results [43], information related to patient treatment as well as control group intervention should be reported [21].

Regarding the reporting quality of methodological items in the CONSORT guidelines, the median MIS was very low at 1, and most trials had insufficient or inadequate information about ‘allocation concealment and implementation’, ‘blinding’, and ‘ITT analysis’. Other published studies also reported similar findings [21, 31, 44–46]. These key methodological items are critical to avoid bias in selection, performance/detection, and attribution. Ultimately, clinical trials with inadequate methodological design can overestimate therapeutic effects [47]. In order to resolve these problems, more researchers involved in clinical trials must be trained in study design and RCT reporting. Additionally, more high-quality research articles must be published in international journals after accurate peer review.

Although there was no significant predictor of improved methodological quality among variables in the regression model, OQS based on the CONSORT guidelines was associated with year of publication. This finding indicates that the mean OQS increased by approximately 0.81 in articles published in successive 5-year periods and improved over time. This finding has also been reported in some previous studies [29, 45], indicating that the application of CONSORT guidelines and the quality of RCT reporting have increased.

### Limitations

Some limitations of this study should also be addressed. First, although it is not difficult to search most studies published in China, it was difficult to obtain the full text of all articles required for this study, as described in Fig. 1. Second, we had difficulty searching for papers published in languages other than Chinese or English. However, most RCTs about trials for scalp acupuncture were reported in Chinese or English [48]. Third, CONSORT and STRICTA were first published in 2001, and there is a high possibility that articles published prior to 2001 may not comply with these guidelines with regard to study design, randomization, and result reporting. Although some articles still do not conform to the guidelines, this situation is gradually improving.

## Conclusions

This study demonstrates the reporting quality of RCTs investigating scalp acupuncture for stroke. Our study demonstrated that the overall quality of reporting on RCTs of scalp acupuncture for stroke was moderate to low. However, the quality of the reporting of key methodological items is particularly lacking. In this field, these findings stress the need to improve methodological quality through increased compliance with the CONSORT and STRICTA guidelines.

## Additional files

Additional file 1: List of RCTs reporting in the scalp acupuncture treatment of stroke (*n* = 63). (DOCX 28 kb)

Additional file 2: Summary of the RCTs reporting in the scalp acupuncture treatment of stroke (*n* = 63). (DOCX 36 kb)

Additional file 3: Measure the overall quality score (OQS) of 13 CONSORT items, a combined key methodological index score (MIS) of 5 CONSORT items, and the OQS of 17 STRICTA items of the randomized control trials of SA for stroke included in this study(*n* = 63). (DOCX 126 kb)

## Abbreviations

CiNii: National institute of informatics scholarly and academic information navigator
CNKI: China national knowledge infrastructure
CONSORT: Consolidated standards of reporting trials
ITT: Intention to treat
KISS: Korean studies information service system
KTKP: Korean traditional knowledge portal
MIS: Methodological index score
NDSL: National digital science library
OQS: Overall quality score
RCT: Randomized controlled trial
STRICTA: Standards for reporting interventions in controlled trials of acupuncture
