# Supplementary material for: Assessing the quality of reports about randomized controlled trials of scalp acupuncture combined with another treatment for stroke
Source: BMC Complement Altern Med. 2017 Sep 6;17:452. doi: 10.1186/s12906-017-1950-6 (PMC5588620; doi:10.1186/s12906-017-1950-6)
Supplement: Supplementary file 1 — List of RCTs reporting in the scalp acupuncture treatment of stroke (n = 63). (DOCX 28 kb) [file 12906_2017_1950_MOESM1_ESM.docx]

**Additional file 1. List of RCTs reporting in the scalp acupuncture treatment of stroke (n=63)**

1. Tae-Sung Cho, In-Suk Son, In-Beohm Park, Snag-Woo Kim, Jung-Chul Seo, Hyoun-Min Youn, Kyung-Jeon Jang, Choon-Ho Song, Chang-Beohm Ahn. Effects of scalp acupuncture on short-term NIHSS and MBI in stroke patients. Journal of Korean Oriental Medicine. 2003, 24(1):65-73

2. LI, Hong, HOU Zhongwei, BAI Yulan, GU Shizhe. Comparison of the Therapeutic Effects among Scalp-, Body.acupuncture and Scalp- plus Body- acupuncture in the Treatment of 230 Cases of Stroke. Acupuncture research. 2006, 31(3):169-172

3. Zhou Shoubang, Lu Dong, Huang Fang, Zhang Wangqiong, Wei Xianping. Effect of Wenshen Huoxue decoction combined with scalp acupuncture on acute cerebral infarction and effect on CRP, UA, FIB. Modern journal of integrated Chinese traditional and western medicine. 2013, 22(10):1027-1029

4. Yu Chang-de, Wu Bing-huang, Zhang Jing, Song Hong-mei, Wang Guo-shu, Yu Zhou. Effect of skull acupuncture and scalp acupuncture on serum vascular endothelial growth factor in the patient of acute cerebral infarction. Chinese acupuncture & moxibustion. 2006, 26(7):466-468

5. Yu Changde, Wu Binghuang, Hong Anhui, Bai Jingyu, Yu Zhou. Changes of Serum MDA Content and Neurological Rehabilitation in Cerebral Infarction Patients Treated with Scalp-acupuncture plus Medication. Acupuncture research. 2004, 29(3):222-225

6. Wang Xuping, Liu Youxiang. Clinical Observation on the Treatment of Acute Cerebral Infarction with Scalp-Acupuncture. World journal of acupuncture-moxibustion. 2001, 11(3):24-27

7. Tang Xi, Tang Cheng-lin, Xu Fang-ming, Xie Hong-wu, Li La-mei, Song Yun-e. Effect of Scalp Acupuncture Combined with Body Acupuncture on Limb Function in Subacute Stroke Patients. Acupuncture research. 2012, 37(6):488-492

8. Wu Tu Hsing, Marta Imamura, Kayleen Weaver, Felipe Fregni, Raymundo S. Azevedo Neto. Clinical Effects of Scalp Electrical Acupuncture in Stroke: A Sham-Controlled Randomized Clinical Trial. The journal of alternative and complementary medicine. 2012, 18(1):341-346

9. Gabriella Hegyi, Gyula P. Szigeti. Rehabilitation of Stroke Patients Using Yamamoto New Scalp Acupuncture: A Pilot Study. The journal of alternative and complementary medicine. 2012, (18(10):971-977

10. Niu Wen-min. Study on mechanism of scalp electro-acupuncture in treating ischemic stroke based on CT localization. Journal of acupuncture and tuina science. 2006, 4(6):333-335

11. Tan Ji-lin, Li Guo-hui. Controlled study on body acupuncture and scalp acupuncture for treatment of ischemic apoplexy. Chinese acupuncture & moxibustion. 2004, 24(6):371-373

12. Liu Yue, Ling Fangming, Cai Gaoning, Lin Xuexia, Long Muheng. Clinical therapeutic effects of electroacupuncture at different acupoints of the head on ischemic apoplexy. Chinese acupuncture & moxibustion. 2004, 24(8):562-564

13. Li Yingkun, Chen Xujun. Study on Scalp Electroacupunture Treatment of Apoplexy and Its Influence on Hemorheology in Ischemic Apoplexy Patients. World journal of acupuncture-moxibustion. 2001, 11(4):18-22

14. Liu Chunhui, Wang Ying. Observation of curative effect of acupuncture therapy plus scalp acupuncture for restoring consciousness and inducing resuscitation in 80 cases of acute apoplexy. Journal of traditional Chinese medicine. 1996, 16(1):18-22

15. Pang Hong. 52 cases of apoplexy treated with scalp acupuncture by the slow-rapid reinforcing-reducing method. Journal of traditional Chinese medicine. 1994, 14(3):185-188

16. Cheng Yan-Hong, Lu Xue-Yuan, Yu Xiao-Gang. Clinical observation on therapeutic effect of three-step acupuncture for the secondary prevention of ischemic cerebral apoplexy. Chinese acupuncture & moxibustion. 2010, 30(4):270-274

17. Shu Changde, Tun Binghuang, Bo Jingyu. Clinical study on needling skull suture plus medicine for treatment of acute cerebral infarction. Chinese acupuncture & moxibustion. 2004, 24(5):329-332

18. Yu Changde, Wu Binghuang, Hong Anhui. Observation on therapeutic effect of scalp acumpuncture plus medicine on early cerebral infarction. Chinese acupuncture & moxibustion. 2003, 23(2):67-69

19. Zhang Lihua, Zhang Shu, Zhang Ailing, Zhang Xia. Effects of Scalp Acupuncture Combined with Motor Rehabilitation Synchronously on Recovery of Upper Limbs Muscle Tension in Acute Infarction Patients. Liaoning Journal of Traditional Chinese Medicine. 2014, 41(12):2676-2678

20. Zhang Bing-hua. Effect of Scalp Acupuncture on the Contents of Serum CRP and Fib in Cerebral Infarction Patients. 2015, 31(2):37-38

21. ZHANG Hong-wei, DAI Xiao-hong, QI Huan, ZHAO Jing. Effect of Scalp Acupuncture Combined with Buyang Huanwu Decoction on Hcy Scale of Patients in the Recovery Stage of Cerebral Infarction. Information on Traditional Chinese Medicine. 2015, 32(3):112-114

22. Dai Jie, Xiang Rong, Chen Yong, Zhang Shiling. The best time window for acute cerebral infarction treated with scalp acupuncture combined with rehabilitation. Modern Journal of Intergrated Traditional Chinese and Western Medicine. 2014, 23(13): 1381-1383

23. ZHANG Shiliang, DAI Jie, TIAN Rui. Observation of scalp therapy combined with exercise imagination and Bobath technique on motor ability and balance func tion of patients with cerebral infarction. Hebei Journal of Traditional Chinese Medicine. 2014, 36(3):341-343

24. XIE Bo, YANG Guilian, LI Mengxian. Observation of Clinical Effects with Scalp Acupuncture and HBO in the Treatment of Cerebral Infarction. Journal of Traditional Chinese Medicine University of Hunan. 2014, 34(7) :51-53,63

25. ZHANG Lihua, WANG Yanjun, ZHANG Shu. Effect of Double Scalp Acupuncture Combined with Rehabilitation Training Synchronously on Acute Infarction Patients with Movement Disturbanc. Journal of Sichuan Traditional Chinese Medicine. 2014, 32(9):152-154

26. ZHANG Shu, ZHANG Li-hua, WANG Yan-jun, ZHANG Ai-ling, ZHANG Xia.

Effects of synchronous treatment of bilateral scalp acupuncture and rehabilitation training on activities of daily life in patients with cerebral infarction at acute phase. Chinese Acupuncture & Moxibustion. 2014, 31(9):837-840

27. WANG Jingjun, JIN Zhangan, LIANG Zuo, CUI Yixin. Effect on the Neurological Deficits of Spastic Hemiplegia after Acute Cerebral Infarction Exerted by the Practice of Zhu Scalp Acupuncture (needle retention required) Coordinated with Limbs Training. Journal of Emergency in Traditional Chinese Medicine. 2013, 22(4):543-544,596

28. YU Chuan, SHEN Bin, XU Yin-ping. Long Retaining Scalp Acupuncture Combined with Tension Balance Acupuncture for Improving the Neurological Deficit of Cerebral Infarction of Patients with Paralysis. Journal of Clinical Acupuncture and Moxibustion. 2013, 29(6):36-38

29. ZHOU Shoubang, LU Dong, WEI Xianping, ZHANG Wangqiong, HUANG Fang. Clinical Study on Wenshenhuoxue Decoction and Scalp Acupuncture in Patients with Acute Cerebral Infarction. Journal of Emergency in Traditional Chinese Medicine. 2012, 21(7):1044-1046

30. YU Xue-ping, YIN Ji-fang, SUN Xiao-wei, ZOU Wei. Effects of Different Intervention Times on Scalp Acupuncture Treatment for Acute Cerebral Infarction. Shanghai Journal of Acupuncture and Moxibustion. 2011, 30(11):730-732

31. LI Chang-fa, JIA Chun-sheng1, LI Xiao-feng1, SHI Jing2, DOU Zhen-zhen, SUN Ping. Effect of Penetrative Needling of Otopoints Combined with Body Acupuncture on Limb Myodynamia and Neurofunction in Patients with Acute Cerebral Infarction. Acupuncture Research. 2010, 35(1):56-60

32. AN Xiangping, XUE Weihua, WANG Yanjun, LI Xiufen, WEI Lifang. Clinical observation of combination of head acupuncture, body acupuncture and rehabilitation training of position on cerebral infarction. Hebei Journal of Traditional Chinese Medicine. 2010, 32(8):1203-1205

33. YE Tian-shen, ZHU Bei-lei, HAN Zhao, GUO Xian-ri, XIE Wen-xia, CHEN Yong, DONG Hai-xin, ZHENG Rui-xian. A control study of magnetic resonance diffusion weighted imaging of patients with acute cerebral infarction and early(《48 hours)intervention with resuscitating and scalp acupuncture therapies(醒脑开窍针刺加头针法). Chinese Journal Of Integrated Traditional And Western Medicine In Intensive And Critical Care. 2008, 15(2):95-97

34. YE Tian-shen, ZHU Bei-lei, XIE Wen-xia, HAN Zhao, CHEN Yong, DONG Hai-xin, Chen Guo-qian. Research of Resuscitating Acupuncture Therapy and Scalp Acupuncture Intervention on Motor Function Recovery in Patients With Acute Cerebralinfarction. Chinese Archives of Traditional Chinese Medicine. 2008, 26(8):1780-1782

35. DENG Shurong, WANG Xianming, ZUO Shupo, LIANG Tao, LI Hongzuo, HE Guangyin. xing nao kai qiao zhen ci jia tou zhen fa dui ji xing nao geng si huan zhe xue qing chao min c- fan ying dan bai shui ping de ying xiang . Clinical Medicine. 2012, 32(10):113-114

36. PI Min, RAO Xiaodan, CAO Xuemei, WU Lixiong, PENG Junhua, LIU Yuansheng, YU Haibo, YANG Zhuoxin. Study on effects of different acupuncture therapies on serum C- reactive protein level in patients with acute cerebral infarction. Journal Of Clinical Acupuncture and Moxibustion. 2007,23(7):1-4

37. YU Chang-de, WU Bing-huang, ZHANG Jing, SONG Hong-mei, WANG Guo-shu, YU Zhou. Effect of skull acupuncture and scalp acupuncture on serum vascular endothelial growth factor in the patient of acute cerebral infarction. Chinese Acupuncture & Moxibustion. 2006, 26(7):466-468

38. WANG Jian, BAI Li. Observations on the curative effect of combined scalp and body acupuncture on cerebral infarction. Shanghai Journal of Acupuncture and Moxibustion. 2006, 25(11):8-9

39. WEI Tiehua, XING Yanli, TANG Qiang. [tou zhen jie he cu jin ji shu dui nao geng si gong neng zhang ai de ping jia](http://caod.oriprobe.com/articles/9494354/tou_zhen_jie_he_cu_jin_ji_shu_dui_nao_geng_si_gong_neng_zhang_ai_de_pi.htm) . Chinese Journal of Rehabilitation Theory & Practice. 2005, 11(7):522-523

40. ZHOU Zuo, WANG Liping, LIU Zuo, BIAN Zuo. Influence of Scalp Acupuncture on Serum Tumor Necrosis Factor in Patients with Acute Cerebral Infarction. Shanghai Journal of Acupuncture and Moxibustion . 2002, 21(1):11-12

41. CHEN Xu-bin, CHEN Li-fang, WANG Yang, ZHOU Yan-xi. Efficacy evaluation of scalp acupuncture and body acupuncture in treating acute cerebral infarction and its impact on vascular endothelial growth factor. Hainan Medical Journal. 2014,25(11):1573-1574,1575

42. WU Xiaojun. [xi yao lian he er zhen tou zhen ti zhen zhi liao nao geng si liao xiao guan cha](http://caod.oriprobe.com/articles/31716589/xi_yao_lian_he_er_zhen__tou_zhen__ti_zhen_zhi_liao_nao_geng_si_liao_xi.htm). Inner Mongol Journal of Traditional Chinese Medicine. 2013(1):39-40

43. YU Dongqiang, ZHANG Xiaoying, HE Dongyong. [tou zhen yun dong liao fa zhi liao ji xing nao geng si 34 li yun dong gong neng zhang ai lin chuang guan cha](http://caod.oriprobe.com/articles/39939425/tou_zhen_yun_dong_liao_fa_zhi_liao_ji_xing_nao_geng_si_34_li_yun_dong_.htm). Medical Journal of Communications. 2013, 27(4):363-365

44. XING Yan-li, TANG Qiang, WEI Tie-hua. Study on Therapeutic Effect of Head Acupuncture Combined with Acceleration Techniques on Stroke Patients Using the SIAS Appraisal Table. Neural Injury and Functional Reconstruction. 2007, 2(1):23-25

45. LI Zhi. [tou zhen yu ti zhen jie he zhi liao ji xing nao geng si liao xiao guan cha.](http://caod.oriprobe.com/articles/9917458/tou_zhen_yu_ti_zhen_jie_he_zhi_liao_ji_xing_nao_geng_si_liao_xiao_guan.htm) Modern Journal of Integrated Traditional Chinese and Western Medicine. 2005,14(13):1723-1724

46. TIAN Lijuan, NA Erbulibahetibieke, YUAN Hongli, NIU Xianglai, ZHOU Zuo. [zhen yao bing yong pei he xi yao zhi liao nao geng si ji xing qi lin chuang guan cha.](http://caod.oriprobe.com/articles/46567308/zhen_yao_bing_yong_pei_he_xi_yao_zhi_liao_nao_geng_si_ji_xing_qi_lin_c.htm)  Journal of Emergency in Traditional Chinese Medicine. 2015,24(8):1479-1480

47. GAO Guoquan, HAO Genlong, LIU Hechun. [dian tou zhen dui ji xing qi nao geng si zhi liao zuo yong de guan cha](http://caod.oriprobe.com/articles/45020354/dian_tou_zhen_dui_ji_xing_qi_nao_geng_si_zhi_liao_zuo_yong_de_guan_cha.htm) . Clinical Journal of Traditional Chinese Medicine. 2014, 26(2):165-166

48. WU Xiangqiong, CHEN Xubin, LIU Yan, YOU Longwu, CHEN Jianxiong, LI Zhuang. Effects of Scalp and Body Acupuncture on TXB2,6-keto-PGF1α Levels in Patients with Acute Cerebral Infarction. Chinese Journal of Traditional Medical Science and Technology. 2014, 21(4):353-354

49. XIONG Jia-wei, NI Guang-xia. Observation of Recovery Period of Acupuncture combined with Medicine in Cerebral Infarction. Journal of Emergency in Traditional Chinese Medicine. 2013,22(6):905-906

50. WU Xiang-qiong, LI Zhuang, YOU Long-wu, CHEN Jian-xiong, LIU Yan. Effects of Serum VEGF on the Patients with Acute Cerebral Infarction by Scalp with Body Acupunc ture. Journal of Clinical Acupuncture and Moxibustion. 2013, 29(7):8-10

51. DENG Shurong, WANG Xianming, ZUO Shupo, TAN Yongming, LIANG Tao, LI Hongzuo, HE Guangyin.

xing nao kai qiao zhen ci jia tou zhen fa zhi liao ji xing nao geng si liao xiao de lin chuang yan jiu. Clinical Medicine. 2012, 32(7):111-112

52. ZHU Guang-qi, HUANG Si-yi. Effect of Xingnaoyinyang Penetration Needling Method on the Level of Ca2+ in Serum of Patients with Acute Cerebral Infarction. Journal of Emergency in Traditional Chinese Medicine. 2011,20(1):9-11

53. CAI Jingzhou, PAN Jinyao. Scalp Acupuncture and Early Blood Vessel Reopening. Shanghai Journal of Acupuncture and Moxibustion. 2002, 21(4):9-10

54. XIE Yun, LIAO Xing-fu, YUAN Yong, ZHAO Zhen-qiang, WU Cui-ping, HUANG Jian-lin, ZOU Bei-lei, SUN Zhao, FU Ying-wen. Effect of different method of scalp acupuncture on VEGF,hs-CRP of patients with cerebral arterial thrombosis. Journal of Hainan Medical University. 2013, 19(2):170-173

55. LIANG Jianqing. [tou zhen ding zuo qian xie xian zhi liao zhong shu xing san cha shen jing tong liao xiao guan cha](http://caod.oriprobe.com/articles/24176114/tou_zhen_ding_zuo_qian_xie_xian_zhi_liao_zhong_shu_xing_san_cha_shen_j.htm). Shanghai Journal of Acupuncture and Moxibustion. 2010, 29(8):528

56. WANG Fan, OU Yanggang, JIA Shaowei. Investigation on effects of acupuncture at scalp motor areas of different sides on cerebral blood flow in the patient of stroke by means of photon emission computed tomography. Chinese Acupuncture & Moxibustion. 2004, 24(5):343-346

57. LI Xiaoning, HUO Huixia, GUAN Zhimin, LIU Shuangling, LIU Huihui. [tou xue cong ci dui ying xiang ji xing nao geng si huan zhe xiang guan yin su de lin chuang yan jiu](http://caod.oriprobe.com/articles/41923208/tou_xue_cong_ci_dui_ying_xiang_ji_xing_nao_geng_si_huan_zhe_xiang_guan.htm). Heilongjiang Medical Journal. 2014, 38(5):503-504

58. GUO Yuhong, ZUO Xiuying, DAI Xiaohong, ZHANG Chunfang. [tou ti zhen liao fa dui nao geng si huan zhe xue jiang tong xing ban zuo an suan shui ping de ying xiang](http://caod.oriprobe.com/articles/27994354/tou_ti_zhen_liao_fa_dui_nao_geng_si_huan_zhe_xue_jiang_tong_xing_ban_z.htm). Acta Chinese Medicine and Pharmacology. 2011, 39(4):87-89

59. HU Zhi-hai, WANG Yi, GENG Li-fang, CUI Li, CHEN Xue-jia, LU Jia, SONG Zheng-yu. Study of Optimization of Different Acupuncture Treatment Protocols for the Early Stage of Ischemic Stroke. Shanghai Journal of Acupuncture and Moxibustion. 2013, 32(10):802-804

60. HUANG Yi, CHEN Zuozuo. [tou zhen yun dong liao fa zhi liao zhong feng 15 li guan cha](http://caod.oriprobe.com/articles/31813342/tou_zhen_yun_dong_liao_fa_zhi_liao_zhong_feng_15_li_guan_cha_头针运动疗法治疗中.htm). Journal of Practical Traditional Chinese Medicine. 2013, 29(1):32-32

61. LI Hong, HOU Zhong-wei, BAI Yu-lan, GU Shi-zhe. Comparison of the Therapeutic Effects among Scalp-, Body-acupuncture and Scalp- plus Body-acupuncture in the Treatment of 230 Cases of Stroke. Acupuncture Research. 2006, 31(3):169-172

62. Ou Yang-Gang. The influence of scalp acupuncture and on somatosensory evoked potential in patients with acute cerebral infarction. Shanghai journal of acupuncture and moxibustion, 2000, 19(4):8-9

63. Shen T and Dong G. Effect of penetration acupuncture of affected side's and bilateral scalp points on TCD in acute cerebral infarction. Shanghai Journal of Acupuncture and Moxibustion. 2002, 21(1):8-10
