# Supplementary material for: Assessing the quality of reports about randomized controlled trials of scalp acupuncture combined with another treatment for stroke
Source: BMC Complement Altern Med. 2017 Sep 6;17:452. doi: 10.1186/s12906-017-1950-6 (PMC5588620; doi:10.1186/s12906-017-1950-6)
Supplement: Supplementary file 2 — Summary of the RCTs reporting in the scalp acupuncture treatment of stroke (n = 63). (DOCX 36 kb) [file 12906_2017_1950_MOESM2_ESM.docx]

**Additional file 2. Summary of the randomized control trials of SA for stroke included in this study(n=63)**

| References | Study design | Eligibility  criteria/Diagnosis | Intervention group  (sample size, male/female,  average age [y]) | Control group (sample size, male/female, average age[y]) | Outcome measure |
| --- | --- | --- | --- | --- | --- |
| Cho et al. 2003 | RCT | CT or MRI | (A) SA (n=12, 5/7, 64.75±7.62) | (B) BA (n=10, 3/7, 66.90±7.62) | • NIHSS  • MBI |
| Li et al. 2006 | RCT | [22, 23]/CT | (A) SA (n=112, 68/44) | (B) BA (n=51, 34/17)  (C) SA with BA (n=67, 49/18) | •Total effective rate(%)  •CT-scanning-shown absorption rate |
| Zhou et al. 2013 | RCT | [22, 23]/CT or MRI | (A) SA with herbal medicine (n=60) | (B) western medication (n=60) | •CRP, UA, FIB  •neurological deficit score  •Total effective rate(%) |
| Yu et al. 2006 | RCT | [23]/CT or MRI | (A)SA with western medication (n=20, 13/7, 63.05±8.37) | (B) skull acupuncture with western medication (n=20,12/8, 63.40±8.72)  (C) western medication (n=20, 14/6, 62.60±9.83) | •VEGF |
| Yu et al. 2004 | RCT | CT or MRI | (A) SA with western medication(n=30, 18/12, 46-74) | (B) western medication(n=30, 19/11, 45-74)  (C)health subject(n=30, 19/11, 49.37±9.03) | • MDA  • ADL  • BI |
| Wu et al. 2001 | RCT | CT | (A) SEA(n=50) | (B) western medication(n=50) | •Total effective rate(%)  •the integral values |
| Wu et al. 2012 | RCT | CT or MRI | (A) SEA (n=35, 50±9.1) | (B) sham-SEA (n=27, 52±8.65) | • BI  • Rankin scale  • NIHSS |
| Tang et al. 2012 | RCT | [23]/CT or MRI | (A) SA (n=55, 27/28, 62.8±8.2) | (B) western medicaition (n=55, 30/25, 64.3±7.7) | • FMA  • NIHSS |
| Gabriella and Gyula 2012 | RCT | [22]/MRI | (A) YNSA with rehabilitation (n=25, 8/17, 58.6±10.4) | (B) rehabilitation (n=25, 10/15, 59.8±9.6) | • BI  • RMI  • VAS |
| Niu and Li 2006 | RCT | [22]/CT or MRI | (A) SA (n=30, 20/10, 61.6±8.3) | (B) SEA (n=30, 19/11, 60.9±9.4) | •Total effective rate |
| Tan and Li 2004 | RCT | [22]/CT or MRI | (A) SA (n=29, 19/10, 64.8±9.35) | (B) BA (n=30, 22/8, 6 4.1±8.7) | •neurological deficit score  •Total effective rate  •FIM |
| LIU Y. et al. 2004 | RCT | [22, 24]  /CT or MRI | (A) SEA with western medication (n=60, 37/23, 62.58±7.39) | (B) BA with western medication (n=60, 36/24, 61.64±7.28) | •TCD  •neurological deficit score  •Total effective rate(%) |
| Li and Chen 2001 | RCT | CT or MRI | (A) SEA (n=93, 65/28) | (B) BA(n=90, 59/31) | •Total effective rate(%)  •change of hemorheology  •change of blood rheology |
| Liu and Wang 1996 | RCT | CT | (A) SA (n=80) | (B) BA (n=25) | •Total effective rate(%) |
| Pang 1994 | RCT | Cerebral spinal fluid or CT | (A) slow-rapid reinforcing-reducing SA with BA (n=52) | (B) flat twisting SA with BA (n=33) | •Total effective rate(%) |
| Cheng et al. 2010 | RCT | [23]/CT or MRI | (A) SA with BA (n=60, 34/26, 65.4±8.6) | (B) oral administration(aspirine) (n=60, 39/21, 63.5±9.8) | •the recurrence rate of ischemic cerebral apoplexy  •neurological deficit score  •Total effective rate(%)  •ADL  •TCM symptom score |
| Yu et al. 2004 | RCT | [22]/CT or MRI | (A) SA with western medication (n=30, 17/13, 53.84±10.82) | (B) western mediciation (n=30, 18/12, 53.28±11.37)  (C) health subject (n=30) | •GMP-140  •neurological deficit score  •Total effective rate(%) |
| Yu et al. 2003 | RCT | [22]/CT or MRI | (A) SA (n=30, 18/12) | (B) western medication (n=30, 19/11)  (C) health subject (n=30) | •MDA  •neurological deficit score  •ADL |
| Zhang L.H. et al. 2014b | RCT | [24]/CT or MRI | (A) SA combined with bobath therapy (n=42, 28/14, 64.5±10.4) | (B) SA with bobath therapy above(n=42, 25/17, 62.4±12.1) | • the period of upper limbs flaccid paralysis  •FMA |
| Zhang B.H. 2015 | RCT | [22]/CT or MRI | (A) SA (n=65, 43/22, 68.1±4.2) | (B) western medication (n=65, 38/27, 63.7±5.1) | •neurological deficit score  •CRP and Fib level |
| Zhang H.W. et al. 2015 | RCT | [22]/CT or MRI | (A) SA with western medication (n=30) | (B) western medication with herbal medicine (n=30) | •Hey scale  •Total effective rate(%) |
| Dia J. et al. 2014 | RCT | [22]/CT or MRI | (A) SA with bobath therapy (n=93) | (B) western medication (n=90) | •FMA  •BADL |
| Zhang S.L. et al. 2014 | RCT | [22]/CT or MRI | (A) SA with bobath therapy (n=23, 13/10, 60.8±9.45) | (B) bobath therapy (n=24, 13/11, 60.8±9.35) | • FMA |
| Xie B. et al. 2014 | RCT | [24]/CT or MRI | (A) SA with western medication and HBO therapy (n=120, 76/44, 65.8±10.3) | (B) western medication (n=120, 74/46, 64.0±10.5) | •Total effective rate(%)  •NIHSS  •BI |
| Zhang L.H. et al. 2014a | RCT | [24]/CT or MRI | (A) SA combined with BA and bobath therapy (n=50, 30/20, 60.5±15.4) | (B) SA with BA and bobath therapy above (n=50, 27/23, 63.4±14.9) | •FMA |
| Zhang S. et al. 2014 | RCT | [24]/CT or MRI | (A) SA at bilateral region with bobath therapy (n=28, 18/10, 64±12) | (B) SA at affected region with bobath therapy (n=29, 16/13, 65±14)  (C) SA at bilateral region and then bobath therapy (n=26, 14/12, 63±13) | •MBI  •ADL  •FMA |
| Wang J.J. et al. 2013 | RCT | CT or MRI | (A) SA with limbs training (n=36, 21/15, 55.34±10.63) | (B) western medication (n=36, 19/17, 56.15±10.16) | •Total effective rate(%)  •neurological deficit score |
| Yu C. et al. 2013 | RCT | [24]/CT or MRI | (A) SA with tension balance acupuncture (n=31, 25/6, 60±1.3) | (B) BA (n=31, 27/4, 61±1.2) | •Total effective rate(%)  •neurological deficit score  •ADL by BI  •level of the muscle tension |
| Zhou S. B. et al. 2012 | RCT | [22, 23]/CT or MRI | (A) SA with Wenshen-huoxue Decoction, western medication herbal medicine and rehabilitation (n=60, 36/24, 65.50±7.38) | (B) western medication with herbal medicine and rehabilitation (n=60, 28/32, 64.2±8.17) | •the change of hemorheology  •the change of blood lipid  •neurological deficit score  •Total effective rate(%) |
| Yu X.P. et al. 2011 | RCT | [22] | (A) SA within 24hour with western medication (n=30, 20/10, 60±1)  (B) SA within3day with western medication (n=30, 18/12, 61±2)  (C) SA within 7day with western medication (n=30, 20/10, 62±2)  (D) SA within 14day with western medication (n=30, 23/7, 62±2) | (E) western medication (n=30, 16/14, 60±1) | •neurological deficit score  •Total effective rate(%) |
| Li C.F. et al. 2010 | RCT | [22]/CT or MRI | (A) SA (n=30, 13/17, 62.4±5.4) | (B) BA (n=30, 16/14, 61.9±4.7)  (C)Ear acupuncture (n=30, 17/13, 62.7±5.4) | •the limbs paralysis |
| An X.P. et al. 2010 | RCT | [23, 24]/CT or MRI | (A) SA with BA and rehabilitation(n=50, 28/22, 61) | (B) BA with rehabilitation (n=50, 29/21, 59) | •Total effective rate(%)  •the change of hemorheology |
| YE T.S. et al. 2008a | RCT | [22]/MRI | (A) SA with western medication (n=20, 66.03±11.38) | (B) western medication (n=20, 67.20±9.30) | •NIHSS  •Total effective rate(%) |
| YE T.S. et al. 2008b | RCT | [22]/CT | (A) SA (n=30, 18/12, 66.03±11.38) | (B) BA (n=30, 16/14, 68.80±6.55)  (C) western medication (n=30, 18/12, 67.20±9.30) | •NIHSS  •MAS  •Ashworth score |
| Deng S. R. et al. 2012 | RCT | [24] | (A) SA with BA (n=168, 90/78, 66±12) | (B) BA with western medication (n=162, 85/77, 68±7) | •NIHSS  •MRS  •hs-CRP |
| Pi M. et al. 2007 | RCT | [22]/CT or MRI | (A) XNKQ acupuncture with western medication (n=32) | (B) SA with BA and with western medication (n=31)  (C) SA with western medication (n=31)  (D) BA with western medication (n=32) | •hs-CRP  •Total effective rate |
| Yu C.D. et al. 2006 | RCT | [22]/CT or MRI | (A) skull acupuncture with western medication (n=20, 12/8, 63.40±8.72) | (B) SA with western medication (n=20, 13/7, 63.05±8.37)  (C) western medication (n=20, 14/6, 62.20±9.83) | •VEGF |
| Wang J. and Bai L. 2006 | RCT | [23]/MRI | (A) SA with BA (n=50, 68) | (B) SA (n=50, 30/20, 65)  (C) BA (n=50, 28/22, 66) | •neurological deficit score  •Total effective rate |
| Wei et al. 2005 | RCT | [22]/CT | (A) SA with rehabilitation by Bobath (n=36, 21/15, 58.81) | (B) SA (n=36, 20/16, 59.44)  (C) Rehabilitation by Bobath (n=36, 22/14, 61.50)  (D) western medication (n=36, 22/14, 62.26) | •SIAS  •ADL  •neurological deficit score  •Total effective rate |
| Zhou et al. 2002 | RCT | [22]/CT or MRI | (A) SA (n=32) | (B) western medication (n=30) | •TNF  •limb function |
| Chen et al. 2014 | RCT | CT | (A) SA with BA (n=60, 32/28, 68±3.6) | (B) western medication (n=60, 29/31, 66±3.5) | •Total effective rate  •VEGF |
| Wu 2013 | RCT | [22]/CT or MRI | (A) SA with BA and Ear acupuncture (n=50, 35/15, 67) | (B) Ear acupuncture (n=50, 37/13, 56.5) | •Total effective rate(%) |
| Gan et al. 2013 | RCT | CT | (A) SA (n=34, 16/18, 65.13±7.72) | (B) BA (n=34, 17/17, 63.48±7.49) | •FMA  •neurological deficit score |
| Xing et al. 2007 | RCT | [22]/CT | (A) SA with western medication and rehabilitation by bobath (n=36, 21/15, 58.81±7.51) | (B) SA with western medication (n=36, 20/16, 59.44±6.31)  (C) Rehabilitation by bobath (n=36, 22/14, 61.50±8.05)  (D) western medication (n=36, 22/14, 62.26±7.82) | •SIAS |
| Li 2005 | RCT | [24] | (A) SA with BA and western medication (n=60) | (B) Scalp point acupuncture with BA and western medication (n=60) | •Total effective rate(%) |
| Tian et al. 2015 | RCT | [22, 23]/CT or MRI | (A) SA with western medication and herbal medication (n=75, 37/38, 63.22±8.68) | (B) SA with western medication and herbal medication (n=75, 35/40, 63.14±8.62) | •GCS  •ADL by BI  •MMSE  •Total effective rate(%) |
| Gao et al. 2014 | RCT | [22] | (A) SEA with western medication (n=35, 17/18, 64.26±13.34) | (B) SA with western medication (n=30, 10/20, 64.09±13.18) | •neurological deficit score  •Total effective rate(%) |
| Wu et al. 2014 | RCT | [22]/CT or MRI | (A) SA with western medication (n=45, 26/19, 62.75±9.63) | (B) western medication (n=42, 24/18, 63.15±8.25) | •TxB2  •6-keto-PGF1α  •TxB2/6-keto-PGF1α |
| Xiang and Ni 2013 | RCT | [22, 23] | (A) SA with BA and western medication (n=30, 67.75±15.91) | (B) SA (n=30, 67.12±13.74)  (C) western medication (n=30, 65.54±13.03) | •Total effective rate(%)  •MBI  •neurological deficit score |
| Wu et al. 2013 | RCT | [22]/CT or MRI | (A) ) SA with BA and western medication (n=45, 26/19, 62.75±9.63) | (B) ) western medication (n=42, 24/18, 63.15±8.25) | •VEGF |
| Deng et al. 2012 | RCT | CT or MRI | (A) SA (n=158, 84/74, 65±12) | (B) BA (n=152, 80/72, 68±7) | •NIHSS  •MRS |
| Zhu and Huang 2011 | RCT | [22] | (A) Xingnaoyinyang Penetration neddling method’s SA (n=20, 14/6, 56.13±11.17) | (B) SA (n=20, 9/11, 59.07±11.49)  (C) western medication (n=20, 14/6, 56.03±8.70) | •Ca2+ level  • neurological deficit score |
| Cai and Pan 2002 | RCT | [22]/CT | (A) SA with herbal medication (n=35) | (B) herbal medication (n=41) | •Total effective rate(%)  •CT absortion rate  •neurological deficit score |
| Xie et al. 2013 | RCT | CT or MRI | (A) twirling method’s SA with western medication (n=50)± | (B) needle-retaining method’s SA (n=50)  (C) needle-embedding therapy (n=50)  (D) western medication (n=50) | •VEGF  •hs-CRP |
| Liang 2010 | RCT | MRI | (A) SA (n=45, 10/35) | (B) BA (n=45, 11/34)± | •Total effective rate(%) |
| Wang et al. 2004 | RCT | [22]/CT or MRI | (A) SA at ipsilateral area (n=13, 9/4, 61.3) | (B) SA at contralateral area (n=10, 8/2, 62.1) | •BFCR |
| Li et al. 2014 | RCT | [22] | (A) SA with western medication (n=30) | (B) western medication (n=30) | •hs-CRP  •homocysteine level |
| Guo 2011 | RCT | [22]/CT or MRI | (A) SA with BA and western medication (n=30, 18/12, 63.56±8.85) | (B) western medication (n=30, 16/14, 62.34±8.54) | •plasma Homocysteine level  •Total effective rate(%) |
| Hu et al. 2013 | RCT | [22, 23] | (A) SA with BA and acupuncture point injection (n=30, 17/13, 63) | (B) SA with BA (n=30, 14/16, 64)  (c) BA (n=30, 16/14, 64) | •NIHSS  •FMA  •ADL |
| Huang and Chen 2013 | RCT | [23] | (A) SA (n=15, 6/9, 55) | (B) BA (n=15, 7/8, 57.5) | •Total effective rate(%) |
| Li et al. 2006 | RCT | [22, 23]/CT | (A) SA with BA (n=67, 49/18) | (B) SA (n=112, 68/44)  (C) BA (n=51, 34/17) | •Total effective rate(%)  •Rank test  •CT scanning absortion rate |
| Ou et al. 2000 | RCT | [22]/CT | (A) SEA by blunt needle (n=24) | (B) SA (n=21) | •SEP |
| Shen and Dong 2002 | RCT | CT or MRI | (A) bilateral scalp point (n=18) | (B) affected side’s point(n=18)  (c) western medication(n=18) | •TCD |

SA, Scalp acupuncture; BA, body acupuncture; SIAN, Standard International Acupuncture Nomenclature proposed by World Health Organization; SEA, scalp electrical acupuncture; YNSA, Yamamoto's New SA; NIHSS, the National Institutes of Health Stroke Scale; MBI, Modified Bathel index score; BI, Bathel index score; ADL, Activities of daily living; FMA, The Fugl-Meyer Assessment; VAS, Visual Analogue Scale; FIM, The Functional Independence Measure scale; CT, computerized tomography; MRI, magnetic resonance imaging; WD, western medication; VEGF, vascular endothelial growth factor; VAS, visual analogue scale; RMI, Rivermead Mobility Index; TCD, Trans Cranial Doppler; GMP-140, granular membrane in platelet; BADL, Basic Activity of Daily Living; HBO, hyperbaric oxygen; Hs-CRP, High-sensitivity C-reactive Protein; VEGF, [Vascular endothelial growth factor;](https://en.wikipedia.org/wiki/Vascular_endothelial_growth_factor) XNKQ acupuncture, Xing Nao Kai Qiao; SIAS, Stroke Impairment Assessment. Set; TNF, Tumor Necrosis Factor; GCS, Glasgow Coma Scale; MMSE, [Mini–Mental State Examination;](https://en.wikipedia.org/wiki/Mini%E2%80%93Mental_State_Examination) 6-k-PGF1α, 6-Keto Prostaglandin F1α; MRS, [Modified Rankin Scale;](https://en.wikipedia.org/wiki/Modified_Rankin_Scale) BFCR, Blood Flow Change rate; SEP, Somato sensory evoked potential

**References in Additional file 2**

22. The Chinese Medical Association of the Fourth National Cerebrovascular Disease Conference. Essentials of diagnosis of all kinds of cerebrovascular diseases. Chin J Neurol. 1996:29:379.

23. Li P, Wu ZZ, Zhang YR, Li BZ, Hua RC, Wei JN, et al. Criterion of diagnosis and therapeutic effect of apoplexy. Journal of Beijing University of Traditional Chinese Medicine. 1996;19:55-6.

24. The Forth National Cerebrovascular Diseases Conference. Classification, diagnostic criteria and evaluation of neurological impairment for stroke patients. Chin J Neurol. 1996;29:381-3.
