# Supplementary material for: Assessing the quality of reports about randomized controlled trials of scalp acupuncture combined with another treatment for stroke
Source: BMC Complement Altern Med. 2017 Sep 6;17:452. doi: 10.1186/s12906-017-1950-6 (PMC5588620; doi:10.1186/s12906-017-1950-6)
Supplement: Supplementary file 3 — Measure the overall quality score (OQS) of 13 CONSORT items, a combined key methodological index score (MIS) of 5 CONSORT items, and the OQS of 17 STRICTA items of the randomized control trials of SA for stroke included in this study(n = 63). (DOCX 126 kb) [file 12906_2017_1950_MOESM3_ESM.docx]

**Additional file 3. Measure the overall quality score (OQS) of 13 CONSORT items, a combined key methodological index score (MIS) of 5 CONSORT items, and the OQS of 17 STRICTA items of the randomized control trials of SA for stroke included in this study(n=63)**

**Table 1**. Measure the overall quality score (OQS) of 13 CONSORT items

| **13 CONSORT items** | **1** | | **2** | | **3** | | **4** | | **5** | | **6** | | **7** | | **12** | | **13** | | **14** | | **17a** | | **18** | | **19** | | **Total score** | |
| --- | --- | --- | --- | --- | --- | --- | --- | --- | --- | --- | --- | --- | --- | --- | --- | --- | --- | --- | --- | --- | --- | --- | --- | --- | --- | --- | --- | --- |
| **Performed reviewer** | **#1** | **#2** | **#1** | **#2** | **#1** | **#2** | **#1** | **#2** | **#1** | **#2** | **#1** | **#2** | **#1** | **#2** | **#1** | **#2** | **#1** | **#2** | **#1** | **#2** | **#1** | **#2** | **#1** | **#2** | **#1** | **#2** | **OQS#1** | **OQS#2** |
| **Cho et al. 2003** | 1 | 1 | 1 | 1 | 0 | 0 | 1 | 1 | 0 | 0 | 1 | 1 | 0 | 0 | 1 | 1 | 0 | 0 | 1 | 1 | 1 | 1 | 0 | 0 | 0 | 0 | **7** | **7** |
| **Li et al. 2006** | 1 | 1 | 0 | 0 | 0 | 0 | 1 | 1 | 1 | 1 | 0 | 1 | 0 | 0 | 1 | 1 | 1 | 1 | 0 | 0 | 1 | 1 | 0 | 0 | 0 | 0 | **6** | **7** |
| **Zhou et al. 2013** | 1 | 1 | 0 | 0 | 0 | 0 | 1 | 1 | 1 | 1 | 1 | 1 | 0 | 0 | 1 | 1 | 1 | 1 | 1 | 1 | 1 | 1 | 0 | 0 | 0 | 0 | **8** | **8** |
| **Yu et al. 2006** | 1 | 1 | 1 | 1 | 0 | 0 | 1 | 1 | 1 | 1 | 1 | 1 | 0 | 0 | 1 | 1 | 1 | 1 | 1 | 1 | 1 | 1 | 0 | 0 | 0 | 0 | **9** | **9** |
| **Yu et al. 2004** | 1 | 1 | 0 | 0 | 0 | 0 | 1 | 1 | 1 | 1 | 1 | 1 | 0 | 0 | 1 | 1 | 1 | 1 | 1 | 1 | 1 | 1 | 0 | 0 | 0 | 0 | **8** | **8** |
| **Wu et al. 2001** | 1 | 1 | 0 | 0 | 0 | 0 | 1 | 1 | 0 | 0 | 0 | 1 | 0 | 0 | 0 | 0 | 1 | 1 | 0 | 0 | 1 | 1 | 0 | 0 | 0 | 0 | **4** | **5** |
| **Wu et al. 2012\** | 1 | 1 | 1 | 1 | 1 | 1 | 0 | 0 | 0 | 0 | 1 | 1 | 0 | 0 | 1 | 1 | 1 | 1 | 0 | 0 | 1 | 1 | 0 | 0 | 1 | 1 | **8** | **8** |
| **Tang et al. 2012** | 1 | 1 | 1 | 1 | 1 | 1 | 1 | 1 | 1 | 1 | 1 | 1 | 0 | 0 | 1 | 1 | 1 | 0 | 1 | 1 | 0 | 1 | 0 | 0 | 0 | 0 | **9** | **9** |
| **Gabriella and Gyula 2012** | 1 | 1 | 1 | 1 | 1 | 1 | 1 | 1 | 1 | 1 | 1 | 1 | 1 | 1 | 1 | 1 | 1 | 1 | 1 | 0 | 1 | 1 | 0 | 0 | 0 | 0 | **11** | **10** |
| **Niu and Li 2006** | 1 | 1 | 0 | 0 | 0 | 0 | 1 | 1 | 1 | 1 | 1 | 1 | 0 | 0 | 1 | 1 | 1 | 1 | 0 | 0 | 0 | 0 | 0 | 0 | 0 | 0 | **6** | **6** |
| **Tan and Li 2004** | 1 | 1 | 0 | 0 | 0 | 0 | 1 | 1 | 1 | 1 | 1 | 1 | 0 | 0 | 1 | 1 | 1 | 1 | 1 | 0 | 1 | 1 | 0 | 0 | 0 | 0 | **8** | **7** |
| **LIU Y. et al. 2004** | 1 | 1 | 0 | 0 | 0 | 0 | 1 | 1 | 1 | 1 | 1 | 1 | 0 | 0 | 1 | 1 | 1 | 1 | 0 | 0 | 1 | 1 | 0 | 0 | 0 | 0 | **7** | **7** |
| **Li and Chen 2001** | 1 | 1 | 0 | 0 | 0 | 0 | 1 | 1 | 1 | 1 | 1 | 1 | 0 | 0 | 0 | 0 | 1 | 1 | 0 | 0 | 1 | 1 | 0 | 0 | 0 | 0 | **6** | **6** |
| **Liu and Wang 1996** | 1 | 1 | 0 | 0 | 1 | 1 | 1 | 1 | 1 | 1 | 0 | 0 | 0 | 0 | 0 | 0 | 1 | 1 | 0 | 1 | 1 | 1 | 0 | 0 | 0 | 0 | **6** | **7** |
| **Pang 1994** | 1 | 1 | 0 | 0 | 0 | 0 | 1 | 1 | 1 | 1 | 1 | 1 | 0 | 0 | 0 | 0 | 1 | 1 | 0 | 0 | 1 | 0 | 0 | 0 | 0 | 0 | **6** | **5** |
| **Cheng et al. 2010** | 1 | 1 | 1 | 1 | 0 | 0 | 1 | 1 | 1 | 1 | 1 | 1 | 0 | 0 | 1 | 1 | 1 | 1 | 1 | 1 | 1 | 1 | 0 | 0 | 0 | 0 | **9** | **9** |
| **Yu et al. 2004** | 1 | 1 | 0 | 0 | 0 | 0 | 1 | 1 | 1 | 1 | 1 | 1 | 0 | 0 | 1 | 1 | 1 | 1 | 1 | 1 | 1 | 1 | 0 | 0 | 0 | 0 | **8** | **8** |
| **Yu et al. 2003** | 1 | 1 | 0 | 0 | 0 | 0 | 1 | 1 | 1 | 1 | 1 | 1 | 0 | 0 | 1 | 1 | 1 | 1 | 1 | 1 | 1 | 0 | 0 | 0 | 0 | 0 | **8** | **7** |
| **Zhang L.H. et al. 2014b** | 1 | 1 | 0 | 0 | 0 | 0 | 1 | 1 | 1 | 1 | 1 | 1 | 0 | 0 | 1 | 1 | 1 | 1 | 1 | 1 | 1 | 1 | 0 | 0 | 0 | 0 | **8** | **8** |
| **Zhang B.H. 2015** | 1 | 1 | 0 | 0 | 0 | 0 | 1 | 1 | 1 | 1 | 1 | 1 | 0 | 0 | 1 | 1 | 1 | 1 | 1 | 1 | 1 | 1 | 0 | 0 | 0 | 0 | **8** | **8** |
| **Zhang H.W. et al. 2015** | 1 | 1 | 1 | 0 | 0 | 0 | 1 | 1 | 0 | 0 | 1 | 1 | 0 | 0 | 1 | 1 | 1 | 1 | 1 | 1 | 1 | 1 | 0 | 0 | 0 | 0 | **8** | **7** |
| **Dia J. et al. 2014** | 1 | 1 | 0 | 0 | 0 | 0 | 1 | 1 | 1 | 1 | 1 | 1 | 0 | 0 | 1 | 1 | 1 | 1 | 1 | 1 | 1 | 1 | 0 | 0 | 0 | 0 | **8** | **8** |
| **Zhang S.L. et al. 2014** | 1 | 1 | 0 | 0 | 0 | 0 | 1 | 1 | 1 | 1 | 1 | 1 | 0 | 0 | 1 | 1 | 1 | 1 | 1 | 1 | 1 | 1 | 0 | 0 | 0 | 0 | **8** | **8** |
| **Xie B. et al. 2014** | 1 | 1 | 0 | 0 | 0 | 0 | 1 | 1 | 1 | 1 | 1 | 1 | 0 | 0 | 1 | 1 | 1 | 1 | 1 | 1 | 1 | 1 | 0 | 0 | 0 | 0 | **8** | **8** |
| **Zhang L.H. et al. 2014a** | 1 | 1 | 0 | 0 | 0 | 0 | 1 | 1 | 1 | 1 | 1 | 1 | 0 | 0 | 1 | 1 | 0 | 0 | 1 | 1 | 1 | 1 | 0 | 0 | 0 | 0 | **7** | **7** |
| **Zhang S. et al. 2014** | 1 | 1 | 0 | 0 | 0 | 0 | 1 | 1 | 1 | 1 | 1 | 1 | 0 | 0 | 1 | 1 | 1 | 1 | 1 | 1 | 1 | 1 | 0 | 0 | 0 | 0 | **8** | **8** |
| **Wang J.J. et al. 2013** | 1 | 1 | 0 | 0 | 0 | 0 | 1 | 1 | 1 | 1 | 1 | 1 | 0 | 0 | 1 | 1 | 1 | 1 | 1 | 1 | 1 | 1 | 0 | 0 | 0 | 0 | **8** | **8** |
| **Yu C. et al. 2013** | 1 | 1 | 1 | 0 | 0 | 0 | 1 | 1 | 1 | 1 | 1 | 1 | 0 | 0 | 1 | 1 | 1 | 1 | 1 | 1 | 1 | 1 | 0 | 0 | 0 | 0 | **9** | **8** |
| **Zhou S. B. et al. 2012** | 1 | 1 | 0 | 0 | 0 | 0 | 1 | 1 | 1 | 1 | 1 | 1 | 0 | 0 | 1 | 1 | 1 | 1 | 1 | 1 | 1 | 1 | 0 | 0 | 0 | 0 | **8** | **8** |
| **Yu X.P. et al. 2011** | 1 | 1 | 1 | 0 | 0 | 0 | 1 | 0 | 0 | 0 | 0 | 0 | 0 | 0 | 1 | 1 | 1 | 1 | 0 | 0 | 1 | 1 | 0 | 0 | 0 | 0 | **6** | **4** |
| **Li C.F. et al. 2010** | 1 | 1 | 1 | 0 | 0 | 0 | 1 | 1 | 1 | 1 | 1 | 1 | 0 | 0 | 1 | 1 | 1 | 1 | 1 | 0 | 1 | 1 | 0 | 0 | 0 | 0 | **9** | **7** |
| **An X.P. et al. 2010** | 1 | 1 | 1 | 0 | 0 | 0 | 1 | 1 | 1 | 1 | 1 | 0 | 0 | 0 | 1 | 1 | 1 | 1 | 1 | 1 | 1 | 1 | 0 | 0 | 0 | 0 | **9** | **7** |
| **YE T.S. et al. 2008a** | 1 | 1 | 0 | 0 | 0 | 0 | 1 | 1 | 1 | 1 | 1 | 1 | 0 | 0 | 1 | 1 | 1 | 1 | 1 | 1 | 1 | 1 | 0 | 0 | 0 | 0 | **8** | **8** |
| **YE T.S. et al. 2008b** | 1 | 1 | 1 | 1 | 0 | 0 | 1 | 0 | 1 | 1 | 1 | 1 | 0 | 0 | 1 | 1 | 1 | 1 | 1 | 1 | 1 | 1 | 0 | 0 | 0 | 0 | **9** | **8** |
| **Deng S. R. et al. 2012** | 1 | 1 | 0 | 0 | 0 | 0 | 1 | 1 | 1 | 1 | 1 | 1 | 0 | 0 | 1 | 1 | 1 | 1 | 1 | 1 | 1 | 1 | 0 | 0 | 0 | 0 | **8** | **8** |
| **Pi M. et al. 2007** | 1 | 1 | 1 | 1 | 0 | 0 | 1 | 1 | 1 | 1 | 1 | 1 | 0 | 0 | 1 | 1 | 1 | 1 | 1 | 1 | 1 | 0 | 0 | 0 | 0 | 0 | **9** | **8** |
| **Yu C.D. et al. 2006** | 1 | 1 | 0 | 0 | 0 | 0 | 1 | 1 | 1 | 1 | 1 | 1 | 0 | 0 | 1 | 1 | 1 | 1 | 1 | 1 | 1 | 1 | 0 | 0 | 0 | 0 | **8** | **8** |
| **Wang J. and Bai L. 2006** | 1 | 1 | 0 | 0 | 0 | 0 | 1 | 1 | 1 | 1 | 0 | 0 | 0 | 0 | 0 | 0 | 1 | 1 | 0 | 0 | 1 | 1 | 0 | 0 | 0 | 0 | **5** | **5** |
| **Wei et al. 2005** | 1 | 1 | 0 | 0 | 0 | 0 | 1 | 1 | 1 | 1 | 1 | 1 | 0 | 0 | 1 | 1 | 1 | 1 | 1 | 1 | 1 | 1 | 0 | 0 | 0 | 0 | **8** | **8** |
| **Zhou et al. 2002** | 1 | 1 | 0 | 0 | 0 | 0 | 1 | 1 | 1 | 1 | 1 | 1 | 0 | 0 | 1 | 0 | 1 | 1 | 1 | 1 | 1 | 1 | 0 | 0 | 0 | 0 | **8** | **7** |
| **Chen et al. 2014** | 1 | 1 | 1 | 1 | 0 | 0 | 1 | 1 | 1 | 1 | 1 | 1 | 0 | 0 | 1 | 1 | 1 | 1 | 1 | 1 | 1 | 1 | 0 | 0 | 0 | 0 | **9** | **9** |
| **Wu 2013** | 1 | 1 | 0 | 0 | 0 | 0 | 1 | 1 | 1 | 1 | 1 | 1 | 0 | 0 | 1 | 1 | 1 | 1 | 1 | 1 | 0 | 0 | 0 | 0 | 0 | 0 | **7** | **7** |
| **Gan et al. 2013** | 1 | 1 | 0 | 0 | 0 | 0 | 1 | 1 | 1 | 1 | 1 | 1 | 0 | 0 | 1 | 1 | 1 | 1 | 0 | 1 | 1 | 1 | 0 | 0 | 0 | 0 | **7** | **8** |
| **Xing et al. 2007** | 1 | 1 | 0 | 0 | 0 | 0 | 1 | 1 | 1 | 1 | 1 | 1 | 0 | 0 | 1 | 1 | 1 | 1 | 1 | 1 | 1 | 1 | 0 | 0 | 0 | 0 | **8** | **8** |
| **Li 2005** | 1 | 1 | 0 | 0 | 0 | 0 | 1 | 1 | 1 | 1 | 0 | 0 | 0 | 0 | 0 | 0 | 1 | 1 | 1 | 0 | 0 | 0 | 0 | 0 | 0 | 0 | **5** | **4** |
| **Tian et al. 2015** | 1 | 1 | 1 | 1 | 0 | 0 | 1 | 1 | 1 | 1 | 1 | 1 | 0 | 0 | 1 | 1 | 1 | 1 | 1 | 1 | 1 | 1 | 0 | 0 | 0 | 0 | **9** | **9** |
| **Gao et al. 2014** | 1 | 1 | 0 | 0 | 0 | 0 | 1 | 1 | 1 | 1 | 0 | 1 | 0 | 0 | 1 | 1 | 1 | 1 | 1 | 1 | 1 | 1 | 0 | 0 | 0 | 0 | **7** | **8** |
| **Wu et al. 2014** | 1 | 1 | 1 | 1 | 0 | 0 | 1 | 1 | 1 | 1 | 1 | 1 | 0 | 0 | 1 | 1 | 1 | 1 | 1 | 0 | 1 | 1 | 0 | 0 | 0 | 0 | **9** | **8** |
| **Xiang and Ni 2013** | 1 | 1 | 0 | 0 | 0 | 0 | 1 | 0 | 1 | 1 | 1 | 1 | 0 | 0 | 1 | 1 | 1 | 1 | 1 | 1 | 1 | 1 | 0 | 0 | 0 | 0 | **8** | **7** |
| **Wu et al. 2013** | 1 | 1 | 1 | 1 | 0 | 0 | 1 | 1 | 1 | 1 | 1 | 1 | 0 | 0 | 1 | 1 | 1 | 1 | 1 | 0 | 1 | 1 | 0 | 0 | 0 | 0 | **9** | **8** |
| **Deng et al. 2012** | 1 | 1 | 0 | 0 | 0 | 0 | 1 | 0 | 1 | 1 | 1 | 1 | 0 | 0 | 1 | 1 | 1 | 1 | 1 | 1 | 1 | 1 | 0 | 0 | 0 | 0 | **8** | **7** |
| **Zhu and Huang 2011** | 1 | 1 | 0 | 0 | 0 | 0 | 1 | 1 | 1 | 1 | 1 | 1 | 0 | 0 | 1 | 1 | 1 | 1 | 1 | 1 | 1 | 1 | 0 | 0 | 0 | 0 | **8** | **8** |
| **Cai and Pan 2002** | 1 | 1 | 0 | 0 | 0 | 0 | 1 | 1 | 0 | 0 | 0 | 0 | 0 | 0 | 0 | 0 | 1 | 1 | 0 | 0 | 1 | 1 | 0 | 0 | 0 | 0 | **4** | **4** |
| **Xie et al. 2013** | 1 | 1 | 1 | 1 | 0 | 0 | 1 | 1 | 1 | 1 | 1 | 1 | 0 | 0 | 1 | 1 | 1 | 1 | 1 | 1 | 1 | 1 | 0 | 0 | 1 | 1 | **10** | **10** |
| **Liang 2010** | 1 | 1 | 0 | 0 | 0 | 0 | 0 | 0 | 1 | 1 | 0 | 0 | 0 | 0 | 0 | 0 | 1 | 1 | 0 | 0 | 0 | 0 | 0 | 0 | 0 | 0 | **3** | **3** |
| **Wang et al. 2004** | 1 | 1 | 1 | 1 | 0 | 0 | 0 | 0 | 1 | 1 | 1 | 1 | 0 | 0 | 1 | 1 | 0 | 0 | 0 | 0 | 1 | 1 | 0 | 0 | 0 | 0 | **6** | **6** |
| **Li et al. 2014** | 1 | 1 | 1 | 1 | 0 | 0 | 1 | 0 | 1 | 1 | 1 | 1 | 0 | 0 | 1 | 1 | 1 | 1 | 0 | 0 | 1 | 1 | 0 | 0 | 0 | 0 | **8** | **7** |
| **Guo 2011** | 1 | 1 | 1 | 1 | 0 | 0 | 1 | 1 | 1 | 1 | 1 | 1 | 0 | 0 | 1 | 1 | 1 | 1 | 0 | 1 | 1 | 1 | 0 | 0 | 0 | 0 | **8** | **9** |
| **Hu et al. 2013** | 1 | 1 | 0 | 0 | 0 | 0 | 1 | 0 | 0 | 1 | 1 | 1 | 0 | 0 | 0 | 1 | 1 | 1 | 1 | 1 | 1 | 1 | 0 | 0 | 0 | 0 | **6** | **7** |
| **Huang and Chen 2013** | 1 | 1 | 0 | 0 | 0 | 0 | 0 | 0 | 1 | 1 | 0 | 0 | 0 | 0 | 0 | 0 | 1 | 1 | 1 | 1 | 0 | 0 | 0 | 0 | 0 | 0 | **4** | **4** |
| **Li et al. 2006** | 1 | 1 | 0 | 0 | 0 | 0 | 1 | 0 | 1 | 1 | 0 | 0 | 0 | 0 | 0 | 1 | 1 | 1 | 0 | 1 | 1 | 1 | 0 | 0 | 0 | 0 | **5** | **6** |
| **Ou et al. 2000** | 1 | 1 | 0 | 0 | 0 | 0 | 0 | 0 | 0 | 1 | 0 | 0 | 0 | 0 | 0 | 0 | 1 | 1 | 0 | 0 | 0 | 0 | 0 | 0 | 0 | 0 | **2** | **3** |
| **Shen and Dong 2002** | 1 | 1 | 1 | 1 | 0 | 0 | 0 | 0 | 1 | 0 | 0 | 0 | 0 | 0 | 0 | 0 | 1 | 1 | 0 | 1 | 1 | 1 | 0 | 0 | 0 | 0 | **5** | **5** |
| **Positive trials** | 63 | 63 | 22 | 17 | 4 | 4 | 57 | 50 | 55 | 56 | 50 | 52 | 1 | 1 | 50 | 51 | 60 | 59 | 44 | 43 | 56 | 54 | 0 | 0 | 2 | 2 | **Max Score** | **11** |
| **Common positive trilas** | 63 | | 17 | | 4 | | 50 | | 54 | | 49 | | 1 | | 49 | | 59 | | 38 | | 53 | | 0 | | 2 | | **Min Score** | **2** |
| **%(n=63)** | 100 | | 27 | | 6 | | 79 | | 86 | | 78 | | 2 | | 78 | | 94 | | 60 | | 84 | | 0 | | 3 | | **Average** | **7** |
| **Cohen's Kappa** | 1.0000  SE = 0.0000 | | 0.8157  SE = 0.0791 | | 1.0000  SE = 0.0000 | | 0.5764  SE = 0.1510 | | 0.7731  SE = 0.1278 | | 0.7945  SE = 0.0995 | | 1.0000  SE = 0.0000 | | 0.8504  SE = 0.0843 | | 0.8489  SE = 0.1499 | | 0.5916  SE = 0.1119 | | 0.7143  SE = 0.1382 | | 1.0000  SE = 0.0000 | | 1.0000  SE = 0.0000 | |  |  |
| **95%CI** | 1.0000 to 1.0000 | | 0.6607 to 0.9707 | | 1.0000 to 1.0000 | | 0.2805 to 0.8723 | | 0.5225 to 1.0237 | | 0.5995 to 0.9894 | | 1.0000 to 1.0000 | | 0.6851 to 1.0156 | | 0.5552 to 1.1427 | | 0.3724 to 0.8109 | | 0.4433 to 0.9853 | | 1.0000 to 1.0000 | | 1.0000 to 1.0000 | |  |  |

CONSORT, Consolidated Standards of Reporting Trials 2010 statement; 1a, ‘Randomized’ in The title or abstract (Study identified as a randomized controlled); in the title or abstract; 2, Background(Adequate description of the scientific background and explanation of rationale); 3, Trial design(Description of trial design such as parallel, factorial including allocation ratio); 4, Participants(Description of the eligibility criteria for participants); 5, Interventions(Details of the interventions intended for each group); 6, Outcomes(Definition of primary and secondary when appropriate outcome measures); 7, Sample size(Description of sample size calculation); 12, Statistical methods(Description of the statistical methods used to compare groups for primary outcomes, subgroup analyses, or adjusted analyses); 13, Flow chart(Details on the flow of participants through each stage of the trials number of patients randomly assigned, receiving intended treatment, completing the protocol and analyzed); 14, Recruitment(Dates defining the periods of recruitment and follow-up); 17, Outcomes and estimation(For each primary and secondary outcome, a summary of results for each group is given, and the estimated effect size and its precision (for example, 95% CI)); 18, Ancillary analyses(Clear statement of whether subgroup/adjusted analyses were prespecified or exploratory); 19, Harms(Description of all important adverse events in each group); OQS, the overall quality score; #1, performed by reviewer 1; #2, performed by reviewer 2; Max score, maximum score in OQS of 13 CONSORT items; Min, minimum score in OQS of 13 CONSORT items; 95%CI, confidence interval

**Table 2.** A combined key methodological index score (MIS) of 5 CONSORT items of the randomized control trials of SA for stroke included in this study(n=63)

| **5 CONSORT items** | **8** | | **9 and 10** | | **11** | | **15** | | **16** | | **Total score** | |
| --- | --- | --- | --- | --- | --- | --- | --- | --- | --- | --- | --- | --- |
| **Performed reviewer** | **#1** | **#2** | **#1** | **#2** | **#1** | **#2** | **#1** | **#2** | **#1** | **#2** | **MIS#1** | **MIS#2** |
| **Cho et al. 2003** | 0 | 1 | 1 | 1 | 1 | 1 | 1 | 1 | 0 | 1 | **3** | **5** |
| **Li et al. 2006** | 1 | 1 | 0 | 0 | 0 | 0 | 1 | 1 | 0 | 0 | **2** | **2** |
| **Zhou et al. 2013** | 0 | 0 | 0 | 0 | 1 | 1 | 0 | 0 | 0 | 0 | **1** | **1** |
| **Yu et al. 2006** | 1 | 1 | 0 | 0 | 1 | 0 | 1 | 1 | 0 | 0 | **3** | **2** |
| **Yu et al. 2004** | 0 | 0 | 0 | 0 | 0 | 0 | 0 | 0 | 0 | 0 | **0** | **0** |
| **Wu et al. 2001** | 0 | 0 | 0 | 0 | 0 | 0 | 0 | 0 | 0 | 0 | **0** | **0** |
| **Wu et al. 2012\** | 0 | 0 | 0 | 0 | 1 | 1 | 1 | 1 | 1 | 1 | **3** | **3** |
| **Tang et al. 2012** | 1 | 1 | 1 | 1 | 1 | 1 | 1 | 1 | 0 | 0 | **4** | **4** |
| **Gabriella and Gyula 2012** | 0 | 0 | 0 | 0 | 1 | 1 | 1 | 1 | 1 | 1 | **3** | **3** |
| **Niu and Li 2006** | 0 | 0 | 0 | 0 | 0 | 0 | 0 | 0 | 0 | 0 | **0** | **0** |
| **Tan and Li 2004** | 1 | 1 | 0 | 0 | 0 | 0 | 0 | 0 | 0 | 0 | **1** | **1** |
| **LIU Y. et al. 2004** | 0 | 1 | 0 | 0 | 0 | 0 | 1 | 0 | 0 | 0 | **1** | **1** |
| **Li and Chen 2001** | 0 | 0 | 0 | 0 | 0 | 0 | 0 | 0 | 1 | 1 | **1** | **1** |
| **Liu and Wang 1996** | 0 | 0 | 0 | 0 | 0 | 0 | 0 | 0 | 0 | 1 | **0** | **1** |
| **Pang 1994** | 0 | 0 | 0 | 0 | 0 | 0 | 0 | 0 | 0 | 0 | **0** | **0** |
| **Cheng et al. 2010** | 1 | 1 | 0 | 0 | 0 | 0 | 1 | 0 | 0 | 0 | **2** | **1** |
| **Yu et al. 2004** | 0 | 1 | 0 | 0 | 0 | 0 | 0 | 0 | 0 | 0 | **0** | **1** |
| **Yu et al. 2003** | 0 | 1 | 0 | 0 | 0 | 0 | 0 | 0 | 0 | 0 | **0** | **1** |
| **Zhang L.H. et al. 2014b** | 1 | 1 | 0 | 0 | 0 | 0 | 0 | 0 | 0 | 0 | **1** | **1** |
| **Zhang B.H. 2015** | 1 | 1 | 0 | 0 | 0 | 0 | 0 | 0 | 0 | 0 | **1** | **1** |
| **Zhang H.W. et al. 2015** | 0 | 1 | 0 | 0 | 0 | 0 | 0 | 0 | 0 | 0 | **0** | **1** |
| **Dia J. et al. 2014** | 0 | 0 | 0 | 0 | 0 | 0 | 0 | 0 | 0 | 0 | **0** | **0** |
| **Zhang S.L. et al. 2014** | 0 | 0 | 0 | 0 | 0 | 0 | 0 | 0 | 0 | 0 | **0** | **0** |
| **Xie B. et al. 2014** | 1 | 1 | 0 | 0 | 0 | 0 | 0 | 1 | 0 | 0 | **1** | **2** |
| **Zhang L.H. et al. 2014a** | 1 | 1 | 0 | 0 | 0 | 0 | 0 | 0 | 0 | 0 | **1** | **1** |
| **Zhang S. et al. 2014** | 1 | 1 | 0 | 0 | 0 | 0 | 0 | 1 | 0 | 0 | **1** | **2** |
| **Wang J.J. et al. 2013** | 0 | 0 | 0 | 0 | 0 | 0 | 1 | 0 | 0 | 0 | **1** | **0** |
| **Yu C. et al. 2013** | 1 | 1 | 0 | 0 | 0 | 0 | 1 | 0 | 0 | 0 | **2** | **1** |
| **Zhou S. B. et al. 2012** | 0 | 0 | 0 | 0 | 1 | 1 | 0 | 0 | 0 | 0 | **1** | **1** |
| **Yu X.P. et al. 2011** | 0 | 0 | 0 | 0 | 0 | 0 | 1 | 1 | 0 | 0 | **1** | **1** |
| **Li C.F. et al. 2010** | 1 | 1 | 0 | 0 | 0 | 0 | 1 | 1 | 0 | 0 | **2** | **2** |
| **An X.P. et al. 2010** | 1 | 1 | 0 | 0 | 0 | 0 | 0 | 0 | 0 | 0 | **1** | **1** |
| **YE T.S. et al. 2008a** | 0 | 0 | 0 | 0 | 0 | 0 | 1 | 1 | 0 | 0 | **1** | **1** |
| **YE T.S. et al. 2008b** | 1 | 1 | 0 | 0 | 0 | 0 | 1 | 1 | 0 | 0 | **2** | **2** |
| **Deng S. R. et al. 2012** | 0 | 0 | 0 | 0 | 0 | 0 | 0 | 0 | 0 | 0 | **0** | **0** |
| **Pi M. et al. 2007** | 1 | 1 | 0 | 0 | 0 | 0 | 0 | 0 | 0 | 0 | **1** | **1** |
| **Yu C.D. et al. 2006** | 1 | 1 | 0 | 0 | 0 | 0 | 1 | 1 | 0 | 0 | **2** | **2** |
| **Wang J. and Bai L. 2006** | 0 | 1 | 0 | 0 | 0 | 0 | 0 | 1 | 0 | 0 | **0** | **2** |
| **Wei et al. 2005** | 0 | 0 | 0 | 0 | 0 | 0 | 0 | 0 | 1 | 1 | **1** | **1** |
| **Zhou et al. 2002** | 0 | 0 | 0 | 0 | 0 | 0 | 0 | 0 | 0 | 0 | **0** | **0** |
| **Chen et al. 2014** | 0 | 0 | 0 | 0 | 0 | 0 | 0 | 0 | 0 | 0 | **0** | **0** |
| **Wu 2013** | 0 | 0 | 0 | 0 | 0 | 0 | 0 | 0 | 0 | 0 | **0** | **0** |
| **Gan et al. 2013** | 1 | 1 | 0 | 0 | 0 | 0 | 0 | 0 | 0 | 0 | **1** | **1** |
| **Xing et al. 2007** | 0 | 0 | 0 | 0 | 0 | 0 | 0 | 0 | 1 | 1 | **1** | **1** |
| **Li 2005** | 0 | 0 | 0 | 0 | 0 | 0 | 0 | 0 | 0 | 0 | **0** | **0** |
| **Tian et al. 2015** | 0 | 0 | 0 | 0 | 0 | 0 | 0 | 0 | 0 | 0 | **0** | **0** |
| **Gao et al. 2014** | 0 | 0 | 0 | 0 | 0 | 0 | 0 | 0 | 0 | 0 | **0** | **0** |
| **Wu et al. 2014** | 0 | 0 | 0 | 0 | 0 | 0 | 0 | 0 | 0 | 0 | **0** | **0** |
| **Xiang and Ni 2013** | 0 | 0 | 0 | 0 | 0 | 0 | 1 | 1 | 0 | 0 | **1** | **1** |
| **Wu et al. 2013** | 0 | 0 | 0 | 0 | 0 | 0 | 1 | 1 | 0 | 0 | **1** | **1** |
| **Deng et al. 2012** | 0 | 0 | 0 | 0 | 0 | 0 | 0 | 0 | 0 | 0 | **0** | **0** |
| **Zhu and Huang 2011** | 0 | 0 | 0 | 0 | 0 | 0 | 0 | 0 | 0 | 0 | **0** | **0** |
| **Cai and Pan 2002** | 0 | 0 | 0 | 0 | 0 | 0 | 0 | 0 | 0 | 0 | **0** | **0** |
| **Xie et al. 2013** | 0 | 0 | 0 | 0 | 0 | 0 | 0 | 0 | 0 | 0 | **0** | **0** |
| **Liang 2010** | 0 | 0 | 0 | 0 | 0 | 0 | 0 | 0 | 0 | 0 | **0** | **0** |
| **Wang et al. 2004** | 0 | 0 | 0 | 0 | 0 | 0 | 0 | 0 | 0 | 0 | **0** | **0** |
| **Li et al. 2014** | 0 | 0 | 0 | 0 | 0 | 0 | 0 | 0 | 0 | 0 | **0** | **0** |
| **Guo 2011** | 1 | 1 | 0 | 0 | 0 | 0 | 0 | 0 | 0 | 0 | **1** | **1** |
| **Hu et al. 2013** | 0 | 0 | 0 | 0 | 0 | 0 | 0 | 0 | 0 | 0 | **0** | **0** |
| **Huang and Chen 2013** | 0 | 0 | 0 | 0 | 0 | 0 | 0 | 0 | 0 | 0 | **0** | **0** |
| **Li et al. 2006** | 1 | 1 | 0 | 0 | 0 | 0 | 0 | 1 | 0 | 0 | **1** | **2** |
| **Ou et al. 2000** | 0 | 0 | 0 | 0 | 0 | 0 | 0 | 0 | 0 | 0 | **0** | **0** |
| **Shen and Dong 2002** | 0 | 0 | 0 | 0 | 0 | 0 | 0 | 0 | 0 | 0 | **0** | **0** |
| **Positive trials** | 19 | 25 | 2 | 2 | 7 | 6 | 17 | 17 | 5 | 7 | **Max Score** | **5** |
| **Common positive trilas** | 19 | | 2 | | 6 | | 13 | | 5 | | **Min Score** | **0** |
| **%(n=63)** | 30 | | 3 | | 10 | | 21 | | 8 | | **Average** | **1** |
| **Cohen's Kappa** | 0.7925  SE = 0.0806 | | 1.0000  SE = 0.0000 | | 0.9143  SE = 0.0850 | | 0.6777  SE = 0.1065 | | 0.8163  SE = 0.1278 | |  |  |
| **95%CI** | 0.6346 to 0.9504 | | 1.0000 to 1.0000 | | 0.7476 to 1.0809 | | 0.4691 to 0.8864 | | 0.5658 to 1.0668 | |  |  |
| CONSORT, Consolidated Standards of Reporting Trials 2010 statement; MIS, a combined key methodological index score; 8, Randomization(Description of the method used to generate the random sequence); 9 and 10, Allocation concealment and implementation(Description of the method used to implement the random allocation sequence assuring the concealment until interventions are assigned); 11, Blinding(Whether or not participants, those administering the interventions, or those assessing the outcomes were blinded to group assignment); 15, Baseline data(An outline of baseline demographic and clinical characteristics of each group); 16, Intent-to-treat analysis(No. of participants in each group included in each analysis and whether it was done by “intention to treat”); #1, performed by reviewer 1; #2, performed by reviewer 2; Max score, maximum score in MIS of 5 CONSORT items; Min, minimum score in MIS of 5 CONSORT items; 95%CI, confidence interval | | | | | | | | | | | | |

**Table 3.** The OQS of 17 STRICTA items of the randomized control trials of SA for stroke included in this study(n=63)

| **17 STRICTA items** | **1a** | | **1b** | | **1c** | | **2a** | | **2b** | | **2c** | | **2d** | | **2e** | | **2f** | | **2g** | |
| --- | --- | --- | --- | --- | --- | --- | --- | --- | --- | --- | --- | --- | --- | --- | --- | --- | --- | --- | --- | --- |
| **Performed reviewer** | **#1** | **#2** | **#1** | **#2** | **#1** | **#2** | **#1** | **#2** | **#1** | **#2** | **#1** | **#2** | **#1** | **#2** | **#1** | **#2** | **#1** | **#2** | **#1** | **#2** |
| **Cho et al. 2003** | 1 | 1 | 1 | 1 | 1 | 1 | 0 | 0 | 1 | 1 | 0 | 0 | 1 | 1 | 1 | 1 | 1 | 1 | 1 | 1 |
| **Li et al. 2006** | 1 | 1 | 1 | 1 | 0 | 0 | 0 | 0 | 1 | 1 | 1 | 1 | 1 | 1 | 0 | 1 | 1 | 1 | 0 | 0 |
| **Zhou et al. 2013** | 1 | 1 | 1 | 1 | 0 | 0 | 0 | 0 | 1 | 1 | 0 | 0 | 1 | 1 | 1 | 1 | 0 | 0 | 1 | 0 |
| **Yu et al. 2006** | 1 | 1 | 1 | 1 | 0 | 0 | 0 | 0 | 1 | 1 | 1 | 0 | 1 | 1 | 0 | 1 | 1 | 1 | 1 | 1 |
| **Yu et al. 2004** | 1 | 1 | 1 | 1 | 0 | 0 | 0 | 0 | 1 | 1 | 1 | 1 | 1 | 0 | 1 | 1 | 1 | 1 | 1 | 1 |
| **Wu et al. 2001** | 1 | 1 | 1 | 1 | 0 | 0 | 1 | 1 | 1 | 1 | 0 | 0 | 0 | 0 | 1 | 1 | 0 | 0 | 0 | 0 |
| **Wu et al. 2012\** | 1 | 1 | 1 | 1 | 1 | 1 | 1 | 1 | 1 | 1 | 1 | 1 | 0 | 0 | 0 | 0 | 0 | 0 | 1 | 1 |
| **Tang et al. 2012** | 1 | 1 | 1 | 1 | 0 | 0 | 0 | 0 | 1 | 1 | 1 | 1 | 1 | 1 | 0 | 0 | 1 | 1 | 1 | 1 |
| **Gabriella and Gyula 2012** | 1 | 1 | 1 | 1 | 0 | 0 | 1 | 1 | 1 | 1 | 1 | 1 | 0 | 0 | 0 | 0 | 1 | 1 | 1 | 1 |
| **Niu and Li 2006** | 1 | 1 | 1 | 1 | 0 | 0 | 0 | 0 | 1 | 1 | 1 | 0 | 0 | 0 | 1 | 1 | 1 | 1 | 1 | 1 |
| **Tan and Li 2004** | 1 | 1 | 1 | 1 | 0 | 0 | 0 | 0 | 1 | 1 | 1 | 1 | 1 | 1 | 1 | 1 | 1 | 1 | 1 | 1 |
| **LIU Y. et al. 2004** | 1 | 1 | 1 | 1 | 0 | 0 | 0 | 0 | 1 | 1 | 0 | 0 | 1 | 1 | 1 | 1 | 1 | 1 | 1 | 1 |
| **Li and Chen 2001** | 1 | 1 | 1 | 1 | 0 | 0 | 1 | 1 | 1 | 1 | 1 | 1 | 1 | 1 | 1 | 1 | 1 | 1 | 1 | 1 |
| **Liu and Wang 1996** | 1 | 1 | 0 | 0 | 0 | 0 | 0 | 0 | 1 | 1 | 1 | 1 | 1 | 1 | 1 | 1 | 1 | 1 | 1 | 1 |
| **Pang 1994** | 1 | 1 | 0 | 0 | 1 | 0 | 0 | 0 | 1 | 1 | 1 | 1 | 1 | 1 | 0 | 0 | 1 | 1 | 1 | 1 |
| **Cheng et al. 2010** | 1 | 1 | 1 | 1 | 0 | 0 | 0 | 0 | 1 | 1 | 1 | 1 | 1 | 1 | 1 | 1 | 1 | 1 | 1 | 1 |
| **Yu et al. 2004** | 1 | 1 | 1 | 1 | 0 | 0 | 0 | 0 | 1 | 1 | 1 | 1 | 1 | 1 | 1 | 1 | 1 | 1 | 1 | 1 |
| **Yu et al. 2003** | 1 | 1 | 1 | 1 | 0 | 0 | 0 | 0 | 1 | 1 | 1 | 1 | 1 | 1 | 1 | 1 | 1 | 1 | 1 | 1 |
| **Zhang L.H. et al. 2014b** | 1 | 1 | 1 | 1 | 0 | 0 | 0 | 0 | 1 | 1 | 0 | 0 | 1 | 1 | 0 | 0 | 1 | 1 | 1 | 1 |
| **Zhang B.H. 2015** | 1 | 1 | 1 | 1 | 0 | 0 | 0 | 0 | 1 | 1 | 1 | 1 | 1 | 1 | 1 | 1 | 1 | 1 | 1 | 1 |
| **Zhang H.W. et al. 2015** | 1 | 1 | 1 | 1 | 0 | 0 | 0 | 0 | 1 | 1 | 1 | 1 | 1 | 1 | 1 | 1 | 1 | 1 | 1 | 1 |
| **Dia J. et al. 2014** | 1 | 1 | 1 | 1 | 0 | 0 | 0 | 0 | 1 | 1 | 1 | 1 | 1 | 1 | 1 | 1 | 1 | 1 | 0 | 0 |
| **Zhang S.L. et al. 2014** | 1 | 1 | 1 | 1 | 0 | 0 | 0 | 0 | 1 | 1 | 1 | 1 | 1 | 1 | 0 | 0 | 1 | 1 | 1 | 1 |
| **Xie B. et al. 2014** | 1 | 1 | 1 | 1 | 0 | 0 | 0 | 0 | 1 | 1 | 0 | 1 | 1 | 1 | 0 | 0 | 1 | 1 | 1 | 0 |
| **Zhang L.H. et al. 2014a** | 1 | 1 | 0 | 1 | 0 | 0 | 0 | 0 | 1 | 1 | 0 | 0 | 1 | 1 | 0 | 0 | 1 | 1 | 1 | 1 |
| **Zhang S. et al. 2014** | 1 | 1 | 1 | 1 | 0 | 0 | 1 | 1 | 1 | 1 | 1 | 0 | 1 | 1 | 0 | 0 | 1 | 1 | 1 | 1 |
| **Wang J.J. et al. 2013** | 1 | 1 | 1 | 1 | 0 | 0 | 0 | 0 | 1 | 1 | 1 | 1 | 0 | 1 | 1 | 1 | 1 | 1 | 1 | 0 |
| **Yu C. et al. 2013** | 1 | 1 | 1 | 1 | 0 | 0 | 0 | 0 | 1 | 1 | 0 | 0 | 1 | 1 | 1 | 1 | 1 | 1 | 1 | 1 |
| **Zhou S. B. et al. 2012** | 1 | 1 | 1 | 1 | 0 | 0 | 0 | 0 | 1 | 1 | 1 | 0 | 1 | 1 | 1 | 1 | 1 | 1 | 1 | 1 |
| **Yu X.P. et al. 2011** | 1 | 1 | 1 | 1 | 0 | 0 | 0 | 0 | 1 | 1 | 0 | 0 | 1 | 1 | 1 | 0 | 1 | 1 | 1 | 1 |
| **Li C.F. et al. 2010** | 1 | 1 | 1 | 1 | 0 | 0 | 0 | 0 | 1 | 1 | 0 | 0 | 1 | 1 | 0 | 0 | 1 | 1 | 1 | 1 |
| **An X.P. et al. 2010** | 1 | 1 | 1 | 1 | 0 | 0 | 0 | 0 | 1 | 1 | 1 | 1 | 1 | 1 | 0 | 0 | 1 | 1 | 1 | 1 |
| **YE T.S. et al. 2008a** | 1 | 1 | 1 | 1 | 0 | 0 | 0 | 0 | 1 | 1 | 1 | 1 | 1 | 1 | 1 | 1 | 1 | 1 | 0 | 0 |
| **YE T.S. et al. 2008b** | 1 | 1 | 1 | 1 | 0 | 0 | 0 | 0 | 1 | 1 | 1 | 1 | 1 | 1 | 1 | 1 | 1 | 1 | 0 | 0 |
| **Deng S. R. et al. 2012** | 1 | 1 | 0 | 0 | 0 | 0 | 0 | 0 | 1 | 1 | 1 | 1 | 1 | 1 | 1 | 1 | 0 | 1 | 0 | 0 |
| **Pi M. et al. 2007** | 1 | 1 | 1 | 1 | 0 | 0 | 0 | 0 | 1 | 1 | 1 | 0 | 1 | 1 | 0 | 0 | 1 | 1 | 0 | 0 |
| **Yu C.D. et al. 2006** | 1 | 1 | 1 | 1 | 0 | 0 | 0 | 0 | 1 | 1 | 1 | 1 | 1 | 1 | 0 | 0 | 1 | 1 | 1 | 1 |
| **Wang J. and Bai L. 2006** | 1 | 1 | 1 | 1 | 0 | 0 | 0 | 0 | 1 | 1 | 1 | 0 | 1 | 1 | 0 | 0 | 1 | 1 | 1 | 1 |
| **Wei et al. 2005** | 1 | 1 | 1 | 1 | 0 | 0 | 0 | 0 | 1 | 1 | 1 | 1 | 1 | 1 | 0 | 0 | 1 | 1 | 1 | 1 |
| **Zhou et al. 2002** | 1 | 1 | 1 | 1 | 0 | 0 | 0 | 0 | 1 | 1 | 1 | 0 | 1 | 1 | 1 | 1 | 1 | 1 | 1 | 1 |
| **Chen et al. 2014** | 1 | 1 | 1 | 1 | 0 | 0 | 0 | 0 | 1 | 1 | 0 | 0 | 1 | 1 | 0 | 0 | 0 | 0 | 1 | 0 |
| **Wu 2013** | 1 | 1 | 1 | 1 | 0 | 0 | 0 | 0 | 1 | 1 | 1 | 1 | 1 | 1 | 1 | 1 | 1 | 1 | 1 | 1 |
| **Gan et al. 2013** | 1 | 1 | 1 | 1 | 0 | 0 | 1 | 1 | 1 | 1 | 1 | 1 | 0 | 0 | 1 | 1 | 1 | 1 | 1 | 0 |
| **Xing et al. 2007** | 1 | 1 | 1 | 1 | 0 | 0 | 1 | 1 | 1 | 1 | 1 | 1 | 1 | 1 | 0 | 0 | 1 | 1 | 1 | 1 |
| **Li 2005** | 1 | 1 | 1 | 1 | 0 | 0 | 0 | 0 | 1 | 1 | 1 | 1 | 1 | 1 | 0 | 0 | 1 | 1 | 1 | 1 |
| **Tian et al. 2015** | 1 | 1 | 1 | 1 | 0 | 0 | 0 | 0 | 1 | 1 | 0 | 0 | 1 | 1 | 0 | 0 | 1 | 1 | 1 | 1 |
| **Gao et al. 2014** | 1 | 1 | 1 | 1 | 0 | 0 | 0 | 0 | 1 | 1 | 1 | 0 | 0 | 0 | 1 | 1 | 1 | 1 | 1 | 1 |
| **Wu et al. 2014** | 1 | 1 | 1 | 1 | 0 | 0 | 0 | 0 | 1 | 1 | 1 | 1 | 1 | 1 | 1 | 1 | 1 | 1 | 1 | 1 |
| **Xiang and Ni 2013** | 1 | 1 | 1 | 1 | 0 | 0 | 0 | 0 | 1 | 1 | 0 | 0 | 1 | 1 | 0 | 0 | 1 | 1 | 1 | 1 |
| **Wu et al. 2013** | 1 | 1 | 1 | 1 | 0 | 0 | 0 | 0 | 1 | 1 | 1 | 1 | 1 | 1 | 0 | 0 | 1 | 1 | 1 | 1 |
| **Deng et al. 2012** | 1 | 1 | 1 | 1 | 0 | 0 | 0 | 0 | 1 | 1 | 1 | 1 | 1 | 1 | 1 | 1 | 1 | 1 | 0 | 0 |
| **Zhu and Huang 2011** | 1 | 1 | 1 | 1 | 0 | 0 | 0 | 0 | 1 | 1 | 1 | 0 | 1 | 1 | 0 | 0 | 1 | 1 | 1 | 1 |
| **Cai and Pan 2002** | 1 | 1 | 1 | 1 | 0 | 0 | 0 | 0 | 1 | 1 | 1 | 0 | 1 | 1 | 0 | 0 | 1 | 1 | 1 | 1 |
| **Xie et al. 2013** | 1 | 1 | 1 | 1 | 0 | 0 | 0 | 0 | 1 | 1 | 1 | 1 | 1 | 1 | 1 | 1 | 1 | 1 | 1 | 1 |
| **Liang 2010** | 1 | 1 | 0 | 0 | 0 | 0 | 0 | 0 | 1 | 1 | 1 | 1 | 0 | 0 | 1 | 1 | 1 | 1 | 1 | 1 |
| **Wang et al. 2004** | 1 | 1 | 1 | 1 | 0 | 0 | 0 | 0 | 1 | 1 | 1 | 1 | 0 | 0 | 0 | 0 | 0 | 0 | 1 | 1 |
| **Li et al. 2014** | 1 | 1 | 1 | 1 | 0 | 0 | 0 | 0 | 1 | 1 | 1 | 0 | 1 | 1 | 1 | 1 | 1 | 1 | 1 | 1 |
| **Guo 2011** | 1 | 1 | 1 | 1 | 0 | 0 | 1 | 1 | 1 | 1 | 1 | 1 | 1 | 1 | 0 | 0 | 1 | 1 | 1 | 1 |
| **Hu et al. 2013** | 1 | 1 | 1 | 1 | 0 | 0 | 0 | 1 | 1 | 1 | 1 | 1 | 1 | 1 | 0 | 0 | 1 | 1 | 1 | 1 |
| **Huang and Chen 2013** | 1 | 1 | 1 | 1 | 0 | 0 | 0 | 1 | 1 | 1 | 1 | 1 | 0 | 0 | 0 | 0 | 1 | 1 | 1 | 1 |
| **Li et al. 2006** | 1 | 1 | 1 | 1 | 0 | 0 | 0 | 0 | 1 | 1 | 0 | 0 | 1 | 1 | 1 | 1 | 1 | 1 | 0 | 0 |
| **Ou et al. 2000** | 1 | 1 | 1 | 0 | 0 | 0 | 1 | 1 | 1 | 1 | 0 | 0 | 1 | 1 | 0 | 0 | 1 | 1 | 1 | 1 |
| **Shen and Dong 2002** | 1 | 1 | 0 | 0 | 0 | 0 | 0 | 1 | 1 | 1 | 1 | 1 | 1 | 1 | 0 | 0 | 1 | 0 | 1 | 1 |
| **합계** | 63 | 63 | 57 | 57 | 3 | 2 | 9 | 12 | 63 | 63 | 48 | 38 | 53 | 53 | 32 | 33 | 57 | 57 | 54 | 49 |
| **positive trilas** | 63 | | 56 | | 2 | | 9 | | 63 | | 37 | | 52 | | 31 | | 56 | | 49 | |
| **%(n=63)** | 100 | | 89 | | 3 | | 14 | | 100 | | 59 | | 83 | | 49 | | 89 | | 78 | |
| **Cohen's Kappa** | 1.0000  SE = 0.0000 | | 0.8158  SE = 0.1282 | | 0.7921  SE = 0.2063 | | 0.8293  SE = 0.0962 | | 1.0000  SE = 0.0000 | | 0.5729  SE = 0.1109 | | 0.8811  SE = 0.0827 | | 0.9047  SE = 0.0537 | | 0.8158  SE = 0.1282 | | 0.7368  SE = 0.1129 | |
| **95%CI** | 1.0000 to 1.0000 | | 0.5646 to 1.0670 | | 0.3878 to 1.1964 | | 0.6407 to 1.0178 | | 1.0000 to 1.0000 | | 0.3554 to 0.7903 | | 0.7190 to 1.0432 | | 0.7994 to 1.0099 | | 0.5646 to 1.0670 | | 0.5155 to 0.9582 | |
| STRICTA, Standards for Reporting Interventions in Controlled Trials of Acupuncture 2010; OQS, the overall quality score; 1a, Style of acupuncture (e.g., Traditional Chinese Medicine, Japanese, Korean, Western medical, Five Element, ear acupuncture, etc.); 1b, Reasoning for treatment provided, based on historical context, literature sources and/or consensus methods, with references where appropriate; 1c, Extent to which treatment was varied; 2a, Number of needle insertions per subject per session(mean and range where relevant); 2b, Names (or location if no standard name) of points used (uni-/bilateral); 2c, Depth of insertion, based on a specified unit of measurement Or on a particular tissue level; 2d, Responses sought (e.g., de qi or muscle twitch response); 2e, Needle stimulation (e.g., manual or electrical); 2f, Needle retention time; 2g, Needle type (diameter, length and manufacturer or material); 3a, Number of treatment sessions; 3b, Frequency and duration of treatment sessions; 4a, Details of other interventions administered to the acupuncture group (e.g., moxibustion, cupping, herbs, exercises, lifestyle advice); 4b, Setting and context of treatment, including instructions to practitioners, and information and explanations to patients; 5, Description of participating acupuncturists (qualification or professional affiliation, years in acupuncture practice, other relevant experience); 6a, Rationale for the control or comparator in the context of the research question, with sources that justify the choice(s); 6b, Precise description of the control or comparator. If sham acupuncture or any other type of acupuncture-like control is used, provide details as for items 1–3 above; #1, performed by reviewer 1; #2, performed by reviewer 2; Max score, maximum score in OQS of 17 STRICTA items; Min, minimum score in OQS of 17 STRICTA items; 95%CI, confidence interval | | | | | | | | | | | | | | | | | | | | |

(continued)

| **17 STRICTA items** | **3a** | | **3b** | | **4a** | | **4b** | | **5** | | **6a** | | **6b** | | **Total Score** | |
| --- | --- | --- | --- | --- | --- | --- | --- | --- | --- | --- | --- | --- | --- | --- | --- | --- |
| **Performed reviewer** | **#1** | **#2** | **#1** | **#2** | **#1** | **#2** | **#1** | **#2** | **#1** | **#2** | **#1** | **#2** | **#1** | **#2** | **OQS#1** | **OQS#2** |
| **Cho et al. 2003** | 1 | 1 | 1 | 1 | 1 | 1 | 1 | 0 | 0 | 1 | 0 | 0 | 1 | 1 | **13** | **13** |
| **Li et al. 2006** | 1 | 1 | 1 | 1 | 0 | 0 | 0 | 0 | 1 | 1 | 0 | 0 | 1 | 1 | **9** | **11** |
| **Zhou et al. 2013** | 1 | 1 | 1 | 1 | 1 | 1 | 0 | 0 | 1 | 1 | 0 | 0 | 1 | 1 | **11** | **10** |
| **Yu et al. 2006** | 1 | 1 | 1 | 1 | 1 | 1 | 0 | 0 | 1 | 0 | 1 | 1 | 1 | 1 | **12** | **12** |
| **Yu et al. 2004** | 1 | 1 | 1 | 1 | 0 | 1 | 0 | 0 | 0 | 0 | 1 | 1 | 1 | 0 | **11** | **11** |
| **Wu et al. 2001** | 1 | 1 | 1 | 1 | 1 | 0 | 0 | 0 | 0 | 0 | 1 | 0 | 1 | 1 | **10** | **8** |
| **Wu et al. 2012\** | 1 | 1 | 1 | 1 | 0 | 0 | 1 | 1 | 1 | 1 | 1 | 1 | 1 | 1 | **12** | **13** |
| **Tang et al. 2012** | 1 | 1 | 1 | 1 | 0 | 0 | 0 | 0 | 1 | 1 | 0 | 0 | 0 | 0 | **9** | **10** |
| **Gabriella and Gyula 2012** | 1 | 1 | 1 | 1 | 1 | 0 | 1 | 1 | 1 | 0 | 1 | 1 | 1 | 1 | **13** | **12** |
| **Niu and Li 2006** | 1 | 1 | 1 | 1 | 1 | 1 | 0 | 0 | 1 | 1 | 1 | 0 | 1 | 1 | **12** | **11** |
| **Tan and Li 2004** | 1 | 1 | 1 | 1 | 0 | 0 | 0 | 0 | 0 | 0 | 1 | 1 | 1 | 1 | **11** | **12** |
| **LIU Y. et al. 2004** | 1 | 1 | 1 | 1 | 1 | 1 | 0 | 0 | 0 | 0 | 0 | 0 | 1 | 1 | **11** | **11** |
| **Li and Chen 2001** | 1 | 1 | 1 | 1 | 0 | 0 | 0 | 0 | 1 | 0 | 1 | 1 | 1 | 1 | **13** | **13** |
| **Liu and Wang 1996** | 1 | 1 | 1 | 1 | 0 | 0 | 0 | 0 | 0 | 0 | 1 | 0 | 0 | 0 | **9** | **9** |
| **Pang 1994** | 1 | 1 | 1 | 1 | 0 | 0 | 0 | 0 | 1 | 0 | 0 | 0 | 1 | 1 | **10** | **9** |
| **Cheng et al. 2010** | 1 | 1 | 1 | 1 | 1 | 1 | 0 | 0 | 1 | 1 | 1 | 0 | 1 | 1 | **13** | **13** |
| **Yu et al. 2004** | 1 | 1 | 1 | 1 | 1 | 1 | 0 | 0 | 1 | 0 | 1 | 1 | 1 | 1 | **13** | **13** |
| **Yu et al. 2003** | 1 | 1 | 1 | 1 | 1 | 1 | 0 | 0 | 1 | 0 | 1 | 1 | 1 | 1 | **13** | **13** |
| **Zhang L.H. et al. 2014b** | 1 | 1 | 1 | 1 | 0 | 1 | 0 | 0 | 1 | 1 | 1 | 1 | 1 | 1 | **11** | **12** |
| **Zhang B.H. 2015** | 1 | 1 | 1 | 1 | 1 | 1 | 0 | 0 | 1 | 1 | 1 | 0 | 1 | 1 | **13** | **13** |
| **Zhang H.W. et al. 2015** | 1 | 1 | 1 | 1 | 1 | 1 | 0 | 0 | 1 | 1 | 0 | 0 | 1 | 1 | **12** | **13** |
| **Dia J. et al. 2014** | 0 | 0 | 1 | 1 | 1 | 1 | 0 | 0 | 1 | 1 | 0 | 0 | 0 | 0 | **9** | **10** |
| **Zhang S.L. et al. 2014** | 1 | 1 | 1 | 1 | 1 | 1 | 0 | 0 | 1 | 1 | 1 | 0 | 0 | 1 | **11** | **12** |
| **Xie B. et al. 2014** | 1 | 1 | 1 | 1 | 0 | 0 | 0 | 0 | 1 | 1 | 1 | 0 | 1 | 1 | **11** | **10** |
| **Zhang L.H. et al. 2014a** | 1 | 1 | 1 | 1 | 0 | 1 | 0 | 0 | 1 | 1 | 1 | 1 | 1 | 1 | **10** | **12** |
| **Zhang S. et al. 2014** | 1 | 1 | 1 | 1 | 0 | 1 | 0 | 0 | 1 | 1 | 1 | 1 | 1 | 1 | **12** | **13** |
| **Wang J.J. et al. 2013** | 1 | 1 | 1 | 1 | 1 | 1 | 0 | 0 | 0 | 0 | 1 | 1 | 1 | 1 | **11** | **12** |
| **Yu C. et al. 2013** | 1 | 1 | 1 | 1 | 1 | 0 | 0 | 0 | 1 | 1 | 1 | 1 | 1 | 1 | **13** | **12** |
| **Zhou S. B. et al. 2012** | 1 | 1 | 1 | 1 | 1 | 1 | 0 | 0 | 0 | 0 | 0 | 0 | 1 | 1 | **11** | **11** |
| **Yu X.P. et al. 2011** | 1 | 1 | 1 | 1 | 1 | 1 | 0 | 0 | 1 | 1 | 1 | 0 | 1 | 1 | **13** | **11** |
| **Li C.F. et al. 2010** | 1 | 1 | 1 | 1 | 0 | 1 | 0 | 0 | 1 | 1 | 1 | 1 | 1 | 1 | **11** | **12** |
| **An X.P. et al. 2010** | 1 | 1 | 1 | 1 | 1 | 1 | 0 | 0 | 1 | 1 | 1 | 1 | 1 | 1 | **12** | **13** |
| **YE T.S. et al. 2008a** | 1 | 1 | 1 | 1 | 1 | 1 | 0 | 0 | 1 | 1 | 1 | 1 | 0 | 1 | **11** | **13** |
| **YE T.S. et al. 2008b** | 0 | 0 | 1 | 1 | 1 | 1 | 0 | 0 | 1 | 1 | 0 | 1 | 1 | 1 | **10** | **12** |
| **Deng S. R. et al. 2012** | 1 | 1 | 1 | 1 | 1 | 0 | 0 | 0 | 0 | 0 | 0 | 0 | 1 | 1 | **8** | **9** |
| **Pi M. et al. 2007** | 1 | 1 | 1 | 1 | 1 | 1 | 0 | 0 | 1 | 1 | 1 | 1 | 1 | 1 | **11** | **11** |
| **Yu C.D. et al. 2006** | 1 | 1 | 1 | 1 | 1 | 1 | 0 | 0 | 1 | 1 | 1 | 1 | 1 | 1 | **12** | **13** |
| **Wang J. and Bai L. 2006** | 1 | 1 | 1 | 1 | 1 | 0 | 0 | 0 | 1 | 1 | 0 | 0 | 1 | 1 | **11** | **10** |
| **Wei et al. 2005** | 1 | 1 | 1 | 1 | 1 | 1 | 0 | 0 | 1 | 1 | 1 | 1 | 1 | 1 | **12** | **13** |
| **Zhou et al. 2002** | 1 | 1 | 1 | 1 | 1 | 1 | 0 | 0 | 1 | 1 | 0 | 0 | 0 | 0 | **11** | **11** |
| **Chen et al. 2014** | 1 | 1 | 1 | 1 | 1 | 1 | 0 | 0 | 0 | 0 | 0 | 0 | 1 | 0 | **9** | **7** |
| **Wu 2013** | 1 | 1 | 1 | 1 | 0 | 0 | 0 | 0 | 0 | 0 | 0 | 0 | 1 | 1 | **10** | **11** |
| **Gan et al. 2013** | 1 | 1 | 1 | 1 | 0 | 0 | 0 | 0 | 0 | 0 | 0 | 1 | 1 | 1 | **10** | **11** |
| **Xing et al. 2007** | 1 | 1 | 1 | 1 | 0 | 1 | 0 | 0 | 1 | 0 | 1 | 1 | 1 | 1 | **12** | **13** |
| **Li 2005** | 1 | 1 | 1 | 1 | 1 | 1 | 0 | 0 | 0 | 0 | 0 | 0 | 0 | 0 | **9** | **10** |
| **Tian et al. 2015** | 1 | 1 | 1 | 1 | 1 | 1 | 0 | 0 | 0 | 0 | 0 | 0 | 1 | 1 | **10** | **10** |
| **Gao et al. 2014** | 1 | 1 | 1 | 1 | 1 | 1 | 0 | 0 | 0 | 0 | 0 | 0 | 0 | 0 | **9** | **9** |
| **Wu et al. 2014** | 1 | 1 | 1 | 1 | 1 | 1 | 0 | 0 | 0 | 0 | 0 | 0 | 0 | 0 | **10** | **11** |
| **Xiang and Ni 2013** | 1 | 1 | 1 | 1 | 1 | 1 | 0 | 0 | 0 | 0 | 0 | 0 | 1 | 1 | **10** | **10** |
| **Wu et al. 2013** | 1 | 1 | 1 | 1 | 1 | 1 | 0 | 0 | 1 | 1 | 0 | 0 | 1 | 1 | **11** | **12** |
| **Deng et al. 2012** | 1 | 1 | 1 | 1 | 1 | 1 | 0 | 0 | 0 | 0 | 0 | 0 | 1 | 0 | **10** | **10** |
| **Zhu and Huang 2011** | 1 | 1 | 1 | 1 | 1 | 1 | 0 | 0 | 0 | 1 | 1 | 0 | 1 | 1 | **11** | **11** |
| **Cai and Pan 2002** | 1 | 1 | 1 | 0 | 1 | 1 | 0 | 0 | 1 | 1 | 0 | 0 | 1 | 0 | **11** | **9** |
| **Xie et al. 2013** | 1 | 1 | 1 | 1 | 1 | 1 | 0 | 0 | 1 | 1 | 0 | 1 | 1 | 1 | **12** | **14** |
| **Liang 2010** | 1 | 1 | 1 | 1 | 0 | 1 | 0 | 0 | 1 | 1 | 1 | 1 | 1 | 1 | **10** | **12** |
| **Wang et al. 2004** | 0 | 1 | 0 | 0 | 0 | 0 | 0 | 0 | 0 | 0 | 1 | 1 | 1 | 0 | **6** | **7** |
| **Li et al. 2014** | 1 | 1 | 1 | 1 | 1 | 1 | 0 | 0 | 1 | 1 | 1 | 0 | 1 | 1 | **13** | **12** |
| **Guo 2011** | 1 | 1 | 1 | 1 | 1 | 1 | 0 | 0 | 1 | 1 | 1 | 0 | 1 | 1 | **13** | **13** |
| **Hu et al. 2013** | 0 | 0 | 0 | 0 | 0 | 0 | 0 | 0 | 1 | 1 | 0 | 0 | 1 | 1 | **8** | **10** |
| **Huang and Chen 2013** | 1 | 1 | 1 | 1 | 0 | 0 | 0 | 0 | 0 | 0 | 0 | 0 | 1 | 1 | **8** | **10** |
| **Li et al. 2006** | 1 | 1 | 1 | 1 | 0 | 0 | 0 | 0 | 1 | 1 | 0 | 0 | 1 | 1 | **10** | **10** |
| **Ou et al. 2000** | 1 | 1 | 0 | 0 | 0 | 0 | 0 | 0 | 1 | 1 | 0 | 0 | 1 | 1 | **10** | **9** |
| **Shen and Dong 2002** | 1 | 1 | 1 | 0 | 0 | 0 | 0 | 0 | 1 | 1 | 0 | 0 | 0 | 0 | **8** | **8** |
| **합계** | 59 | 60 | 60 | 58 | 40 | 42 | 3 | 2 | 42 | 37 | 33 | 25 | 53 | 50 | **Max score** | **14** |
| **positive trilas** | 60 | | 58 | | 35 | | 2 | | 35 | | 22 | | 48 | | **Min score** | **6** |
| **%(n=63)** | 95 | | 92 | | 56 | | 3 | | 56 | | 35 | | 76 | | **Average** | **11** |
| **Cohen's Kappa** | 0.8489  SE = 0.1499 | | 0.7342  SE = 0.1850 | | 0.5814  SE = 0.1087 | | 0.7921  SE = 0.2063 | | 0.6966  SE = 0.0936 | | 0.5599  SE = 0.1037 | | 0.6291  SE = 0.1322 | |  |  |
| **95%CI** | 0.5552 to 1.1427 | | 0.3717 to 1.0967 | | 0.3683 to 0.7945 | | 0.3878 to 1.1964 | | 0.5131 to 0.8801 | | 0.3566 to 0.7632 | | 0.3700 to 0.8882 | |  |  |
| STRICTA, Standards for Reporting Interventions in Controlled Trials of Acupuncture 2010; OQS, the overall quality score; 1a, Style of acupuncture (e.g., Traditional Chinese Medicine, Japanese, Korean, Western medical, Five Element, ear acupuncture, etc.); 1b, Reasoning for treatment provided, based on historical context, literature sources and/or consensus methods, with references where appropriate; 1c, Extent to which treatment was varied; 2a, Number of needle insertions per subject per session(mean and range where relevant); 2b, Names (or location if no standard name) of points used (uni-/bilateral); 2c, Depth of insertion, based on a specified unit of measurement Or on a particular tissue level; 2d, Responses sought (e.g., de qi or muscle twitch response); 2e, Needle stimulation (e.g., manual or electrical); 2f, Needle retention time; 2g, Needle type (diameter, length and manufacturer or material); 3a, Number of treatment sessions; 3b, Frequency and duration of treatment sessions; 4a, Details of other interventions administered to the acupuncture group (e.g., moxibustion, cupping, herbs, exercises, lifestyle advice); 4b, Setting and context of treatment, including instructions to practitioners, and information and explanations to patients; 5, Description of participating acupuncturists (qualification or professional affiliation, years in acupuncture practice, other relevant experience); 6a, Rationale for the control or comparator in the context of the research question, with sources that justify the choice(s); 6b, Precise description of the control or comparator. If sham acupuncture or any other type of acupuncture-like control is used, provide details as for items 1–3 above; #1, performed by reviewer 1; #2, performed by reviewer 2; Max score, maximum score in OQS of 17 STRICTA items; Min, minimum score in OQS of 17 STRICTA items; 95%CI, confidence interval | | | | | | | | | | | | | | | | |
